# Amino acids for the prevention of mortality and morbidity in preterm infants: a systematic review and network meta-analysis

**DOI:** 10.1038/s41598-022-21318-w

**Published:** 2022-10-31

**Authors:** Xiaoqin Wang, Behnam Sadeghirad, Rebecca L. Morgan, Dena Zeratkaar, Yaping Chang, Holly N. Crandon, Rachel Couban, Farid Foroutan, Ivan D. Florez

**Affiliations:** 1 Michael G. DeGroote Institute for Pain Research and Care, McMaster University, Hamilton, Canada; 2 Department of Health Research Methods, Evidence, and Impact (HEI), McMaster University, Hamilton, Canada; 3 Department of Anesthesia, McMaster University, Hamilton, ON Canada; 4 OrthoEvidence, Burlington, Canada; 5 Faculty of Health Sciences, McMaster University, Hamilton, Canada; 6 Ted Rogers Centre for Heart Research, Peter Munk Cardiac Centre, Toronto, Canada; 7 Department of Pediatrics, University of Antioquia, Calle 67 No. 53-108, Medellin, Colombia; 8 School of Rehabilitation Science, McMaster University, Hamilton, Canada; 9 Pediatric Intensive Care Unit, Clínica Las Americas, Medellin, Colombia

**Keywords:** Paediatric research, Outcomes research, Gastrointestinal diseases, Infectious diseases

## Abstract

To determine the effectiveness and safety of amino acids in preventing the mortality and morbidity among preterm infants. We conducted a systematic review and network meta-analysis. We searched MEDLINE, EMBASE, Web of Science, CINAHL, Scopus, Cochrane, and Google Scholar, and grey literature, from databases inception to January 2021. We included randomized trials that evaluated any amino acids on preterm or low-birth weight infants. We performed frequentist pairwise and network meta-analyses and used the GRADE methodology to assess the certainty of the evidence and provide a summary of the results.We included 18 trials (3702 infants). Low certainty evidence showed that there seems to be no benefit for arginine, glutamine, or N-acetylcysteine in reducing all-cause mortality. Oral arginine likely results in reduction of necrotizin enterocolitis (NEC) stage ≥ II (OR 0.48; 95% CI 0.26–0.90; moderate certainty). Oral glutamine may reduce the likelihood of developing late-onset sepsis (LOS) compared to placebo (OR 0.62; 95% CI 0.47–0.82; low certainty); and likely reduces time to reach full enteral feeding (MD = − 2.63 days; 95% CI − 4.99 to − 0.27; moderate certainty). Amino acids may have no effect on mortality. Oral arginine may reduce severe NEC, and oral glutamine may reduce LOS and the time to reach full feeding.

**Systematic review registration**: PROSPERO registration number: CRD4201603873.

## Introduction

Preterm birth is the leading cause of neonatal death globally, accounting for 15.9% of the mortality among neonates^1,2^. Necrotizing enterocolitis (NEC) is the most serious gastrointestinal complication in preterm infants^3^, and it is among the leading causes of mortality and morbidity in neonatal intensive care units (NICUs)^4,5^. NEC-related mortality has been reported to range from 16 to 42%, depending on gestational age and birth weight^6^. Currently, there is no effective treatment for NEC and the surgical management is associated with very high mortality^7,8^. Late-onset sepsis (LOS) is also a common complication in preterm infants representing a significant healthcare burden in the NICUs worldwide. Given its high incidence, significant morbidity (including long-term consequences on growth and development), and mortality, implementing efforts to reduce the infection rates is a priority in neonatal care^9,10^.

Amino acids are essential components of parenteral nutrition. Supplementing amino acids such as glutamine, arginine, and N-acetylcysteine may help modulating the pathophysiology of NEC and LOS by their anti-inflammatory, anti-apoptosis, and antioxidant effects^11–13^. Different amino acids supplementation through different administration routes have been studied in preterm infants for the prevention of NEC and LOS. Two Cochrane reviews by Shah et al.^14^ and Moe-Byrne et al.^15^ have summarized the evidence of the effectiveness and safety of the supplementation of arginine and glutamine, respectively, in preterm infants. Shah et al. found that arginine may reduce the incidence of NEC, while Moe-Byrne did not find enough evidence to support the supplementation of glutamine. In 2018, a review narratively summarized the evidence from 15 RCTs that evaluated the effectiveness of amino acids supplementation for preventing NEC^16^. Authors found that some studies showed benefit for amino acids supplementation in preventing NEC or LOS, while others might have no benefit.

Given the contradictory evidence on the effects of amino acids in conferring benefits to preterm infants and a lack of comparative analysis on the effects of different types of amino acids**,** we aimed to conduct a systematic review and network meta-analysis (NMA) of randomized trials (RCTs) to determine the comparative effectiveness and safety of amino acids supplementation for preventing the mortality and morbidity among preterm infants.

## Methods

This systematic review was registered with PROSPERO (CRD4201603873) and a full protocol was published in an open-access journal^17^. We followed the recommendations provided by the PRISMA-NMA (Preferred Reporting Items for Systematic Reviews and Meta-Analyses) extension in reporting our review^18^.

### Data sources

Based on the search strategy from the protocol, we systematically searched MEDLINE, EMBASE, Science Citation Index Expanded and Social Sciences Citation Index, CINAHL, Scopus, Cochrane Central Register of Controlled Trials, and Google scholar from inception to January 10th, 2021, without any language restrictions. We performed grey literature searches through trial registries (Clinicaltrials.gov and WHO Clinical Trials Registry Platform). The lists of references of the eligible trials and relevant reviews were scanned for any additional eligible trial. Supplementary table 1 provides our search strategy.

### Study selection

We included RCTs enrolling preterm (gestational age < 37 weeks) and/or low birth weight (birth weight < 2500 g) infants randomized to a preventive administration of any amino acid compared to an alternative intervention, placebo, or no treatment. We included studies of any amino acid (including but not limited to glutamine, arginine, and N-acetyl cysteine) with any dose, regimen, frequency, or route of administration. Our outcomes of interest were all-cause mortality, severe NEC (stage ≥ II—Bell's criteria), culture-proven LOS, NEC-related mortality, length of hospitalization (days), time to reach full enteral feeds (days), feed intolerance, weight at 37 weeks’ postnatal age or at discharge, and adverse events.

Pairs of reviewers (XW, DZ, RM, YC, FF, IF) independently and in duplicate screened the titles and abstracts to assess their eligibility. Potentially eligible studies were reviewed in full text. Reviewers (XW, DZ, RM, YC, FF, IF) independently and in duplicate reviewed the eligible full texts. Reviewers resolved any discrepancies through discussion, or consultation with a third reviewer (BS) when needed. We tried to contact authors of included studies during data extraction and risk of bias assessment for missing information.

### Data extraction and risk of bias assessment

Pairs of reviewers (XW, DZ, RM, YC, FF, IF) independently and in duplicate extracted the data and reached consensus through discussion or consultation with a third reviewer (BS) in a piloted format. We collected the basic characteristics of included studies (study design, year, duration of follow-up, sample size), population (gestational age, birth weight, enteral nutrition, and relevant perinatal history), interventions and comparisons (doses, frequency, and regimens), and outcomes (number of events, mean and standard deviation or standard errors). We applied methods proposed in Cochrane Handbook and Hozo et al. to impute mean and standard deviation when median, range, and sample size were reported^19^. For studies published in duplicate or studies that used data from a similar study population in different publications in part or full, we extracted data from the publication with the most complete dataset.

Pairs of reviewers (XW, DZ, RM, YC, FF, IF) assessed the risk of bias independently and in duplicate, and resolved any discrepancies through discussion, or adjudication by a third reviewer (BS). We used a modified Cochrane risk of bias instrument that addresses the following potential sources of bias: random sequence generation, allocation concealment, blinding study participants (infants’ parents in our study), personnel and outcome assessors, and incomplete outcome data. Responses including “definitely yes” or “probably yes” (considered as low risk of bias), or “definitely no” or “probably no” (considered as high risk of bias) were used rather than the standard response options (high, low, or unclear risk of bias)^20,21^.

### Data synthesis and statistical methods

We performed frequentist pairwise random-effects meta-analyses for all direct comparisons. For dichotomous outcomes, we calculated odds ratio (OR), and for continuous outcomes the weighted mean diffearence (WMD), and their corresponding 95% confidence intervals (95% CIs). We used the Q-statistic and I^2^ statistic to measure the statistical heterogeneity in each direct comparison^22^. The I^2^ measures the percentage of variation across studies due to heterogeneity rather than chance^23^. We used the I^2^ value of each direct comparison to evaluate the presence of inconsistency when assessing the certainty of the evidence as described below in the ‘Rating the certainty of the evidence’ subheading. For this purpose, we used a threshold of 50% to define significant inconsistency and rate down the evidence.

A geometry plot was used to present all the available direct comparisons per outcome, in which each node represents one intervention. For those amino acids with different administration forms (e.g., oral, intravenous-i.v.) we separated the evidence in different nodes.

We performed the frequentist random-effects NMA to synthesize the available evidence using the methodology of multivariate meta-analysis assuming a common heterogeneity parameter^24,25^. We evaluated transitivity by inspection and analysis of the clinical similarities between direct comparisons that informed indirect evidence. For this purpose, we considered the following variables: birth weight, gestational age, mean APGAR scores, and percentage of infants born by C-section. We evaluated the presence of incoherence (also called network inconsistency) by comparing direct evidence with indirect evidence using the node splitting approach^22,26^. We also assessed the evidence of incoherence in the entire network using the design-by-treatment model^27^. We calculated ranking probabilities, mean ranks, and rankograms as well as SUCRA values (the Surface Under the Cumulative Ranking curve), for all outcomes.

We performed meta-regression using the Knapp-Hartung modification of the variance regardless of observed heterogeneity to assess the effect modification of gestational age, birth weight and percent infants delivered by C-section^28^. In sparse networks, heterogeneity estimation across the network using contrast-based random-effects model can have strange results which results in spuriously wide confidence interval (i.e., 95% CI of the network estimates were wider than those of the direct estimates or the indirect estimates)^29^. In such cases, we performed fixed-effect model NMA and reported the results of random-effects model as sensitivity analysis in the supplementary files. We planned to assess small-study effect using funnel plots when 10 studies or more were available for the direct comparisons.

### Rating the certainty of the evidence

We used the Grading of Recommendations Assessment, Development, and Evaluation (GRADE) approach to assess the certainty of the evidence for effect estimates from direct, indirect, and NMA evidence for each outcome^29–36^. Initially, we rated the certainty in direct estimates according to the traditional GRADE guidance by considering risk of bias, inconsistency, indirectness, imprecision, and publication bias. Then, we rated the certainty in indirect estimates, starting with the lowest ratings of the direct comparisons contributing to the dominant first-order loop with further rating down, when necessary, for intransitivity. NMA estimate certainty started as the higher of the direct and indirect evidence; however, the relative contribution of direct and indirect evidence to the network estimate was considered when rating the certainty. If incoherence was detected in a specific loop or comparison, we further rated down the certainty of the network estimates.

### GRADE approach to summarize results from NMA

To optimize the interpretation of the findings, we applied the GRADE approach for drawing conclusions from NMAs using a minimally contextualized framework^37^. This approach allows us to categorize the interventions—from the most effective to the least effective—based on the effect estimates obtained from the NMA and their associated GRADE certainty of evidence. For each outcome, we created groups of interventions as follows: Category 0, the reference intervention (placebo) and interventions with no evidence of difference compared to placebo (i.e. 95%CIs include the null value) which we refer to as “among the least effective”; Category 1, interventions superior to placebo, but not superior to any other of the intervention(s) superior to placebo, inferior to the most effective, but superior to the least effective”; category 3, interventions that proved superior to at least one category 2 intervention, termed “among the most effective”. We then divided all categories into two groups: those with moderate- or high- certainty evidence relative to placebo, and those with low- or very low- certainty evidence relative to placebo^37^.

## Results

**Figure 1:**
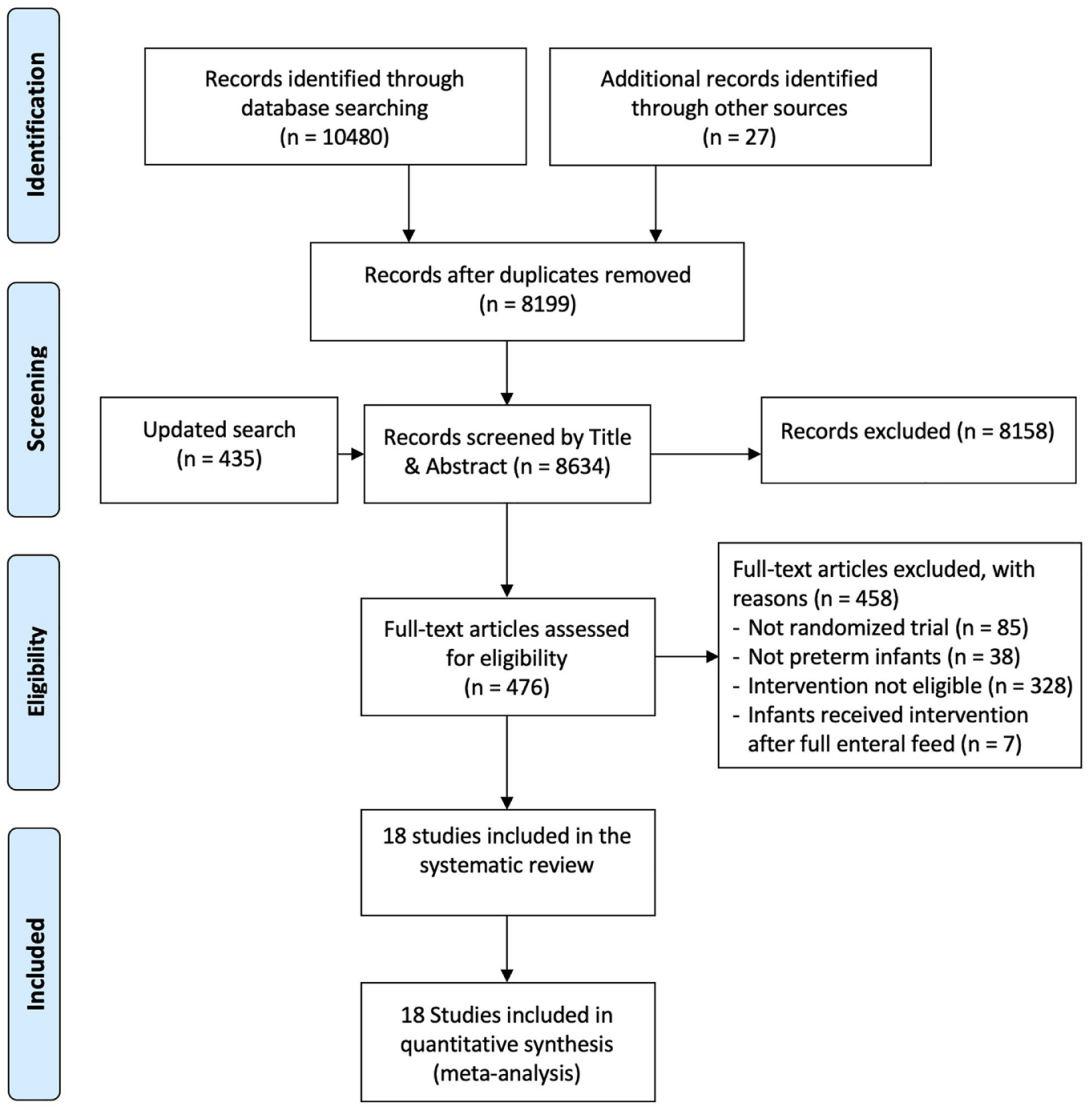
PRISMA Flow diagram of study selection.

**Figure 2:**
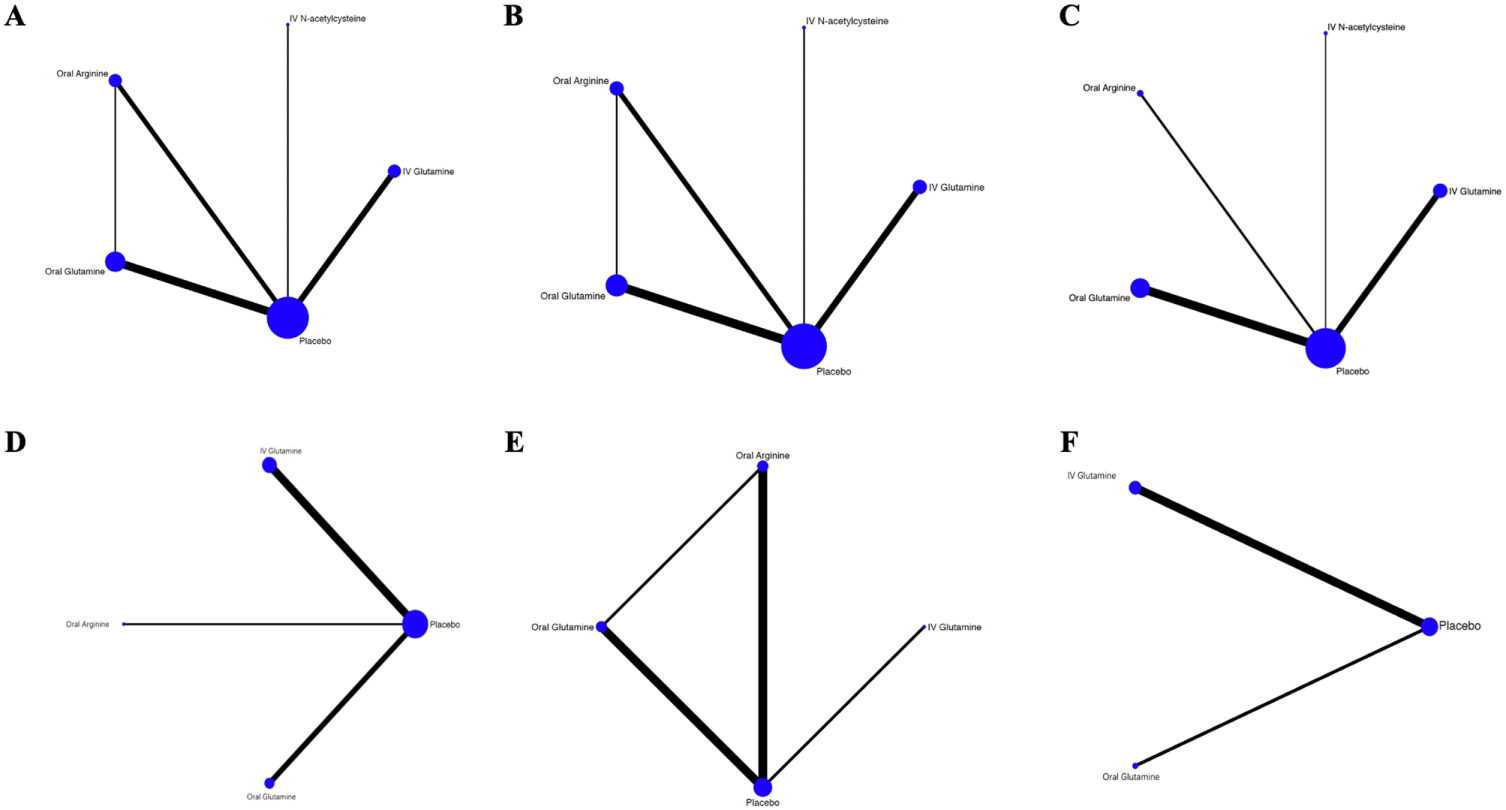
Network meta-analysis plots. (**A**) All-cause mortality, (**B**) severe NEC, (**C**) Late Onset Sepsis, (**D**) Length of hospitalization, (**E**) NEC-related mortality, (**F**) Time to achieve full enteral feeding.

We identified 8634 titles and abstract through our searches, of which 476 full-texts articles were screened for eligibility. We included 18 randomized trials involving 3702 infants^38–55^. Figure 1 provides the details of the study selection process. Across the included trials, the median of average weight and gestational age were 1089.4 g (interquartile range (IQR) 912.5, 1256.1) and 29.0 weeks (IQR 27.4, 29.6), respectively. Of the 18 included studies, seven studies compared oral glutamine with placebo, six trials compared intravenous (i.v.) glutamine with placebo, three compared oral arginine with placebo, one compared i.v. N-acetylcysteine with placebo, and one three-arm trial, comparing oral arginine, oral glutamine and placebo. Table 1 summarizes study characteristics and Fig. 2 presents network of treatments across outcomes.

**Table 1:** Characteristics for included studies.

Study ID	BW (gr)	GA (weeks)	Apgar score M5	%C-Sec	% SGA	% MM fed / %FM fed	Interventions (# patients)	Control (# patients)	Treatment duration (weeks)	Outcomes
Ahola et al.^38^	778	26.4	7.6	58.6	31.2	NR	i.v. N-acetylcysteine (194)	Placebo (197)	0.86	ACM, NEC, LOS
Amin et al.^39^	953	27.5	7.5	NR	7.9	NR	Oral Arginine (75)	Normal saline (77)	4	ACM, NEC, LOS, NRM, WT
Bober-Olesinska and Kornacka^40^	950	28	6	70.9	-	NR	i.v. Glutamine (25)	No glutamine (30)	1.4	ACM, NEC, LOS, HOS
El-Shimi et al.^41^	1400	31.4	8	84	40	6.7/99.3	Arm1: Oral Arginine (25) Arm2: Oral Glutamine (25)	No glutamine or arginine (25)	4	ACM, NEC, NRM
Korkmaz et al.^42^	1241.8	29.6	7.1	85.5	0	NR	Oral Glutamine (36)	Placebo (33)	16	WT, TRFF
Lacey et al.^43^	805.5	26	6.3	68.2	NR	NR	i.v. Glutamine (22)	parenteral nutrition without glutamine (22)	> 1	NEC, LOS, HOS, TRFF
Maamouri et al.^54^	1299.1	29.2	NR	39.1	NR	NR	Oral Glutamine (52)	Routine care (53)	4	NEC, LOS, HOS
Memisoglu et al.^55^	1072.1	29.7	8	75	28.8	NR	Oral Arginine (27)	Distilled water (25)	1	NEC, LOS, HOS, WT
Mohamad Ikram et al.^44^	2185.8	NR	NR	46.4	NR	NR	i.v. Glutamine (76)	Standard parenteral nutrition (78)	NR	ACM, NEC, LOS, NRM, HOS, TRFF
Neu et al.^45^	951.4	27.4	NR	NR	NR	0/100	Oral Glutamine (35)	No glutamine (33)	4	ACM, NEC, LOS
Pawlik et al.^46^	1106.7	29.1	NR	NR	NR	NR	Oral Glutamine (50)	Placebo (56)	NR	ACM, NEC, LOS, NRM, FIT, HOS
Poindexter et al.^47^	769	26	NR	NR	16.1	NR	i.v. Glutamine (721)	No glutamine (712)	17	ACM, NEC, LOS, FIT, HOS, TRFF
Polycarpou et al.^48^	1146.8	29	8	74.7	NR	14.5/85.5	Oral Arginine (40)	5% glucose (43)	4	ACM, NEC, NRM
Sevastiadou et al.^49^	1304.8	30.3	NR	87.1	NR	0/100	Oral Glutamine (51)	Glucose polymer (50)	4	ACM, NEC, LOS
Thompson et al.^50^	891	27.4	NR	NR	NR	NR	i.v. Glutamine (17)	No glutamine (18)	NR	ACM, NEC, LOS, HOS, TRFF
van den Berg et al.^51^	1170.2	29	NR	53.9	NR	28.4/NR	Oral Glutamine (52)	Alanine in breast milk or formula (50)	4	ACM, NEC, LOS, HOS, TRFF
Vaughn et al.^52^	900	27	8	69	11.2	NR	Oral Glutamine (314)	Sterile water with no added glutamine (335)	4	ACM, NEC, LOS, NRM, FIT, HOS, WT
Wang et al.y^53^	1260.9	30.9	NR	NR	NR	NR/100	i.v. glutamine (13)	No glutamine (15)	1	ACM, HOS, TRFF

BW-birth weight, GA-gestational age, Apgar M5- APGAR score at 5-min, %C-Sec-percent infants delivered by caesarean, % SGA- percent infants for small gestational age, % MM fed- percent infants exclusively fed with mother’s milk, %FM fed- percent infants exclusively fed with formula milk. NEC: Necrotizing enterocolitis; ACM: All-cause mortality; LOS: Culture-proven Late-onset sepsis, TRFF: Time to reach full enteral feeding, HOS: Duration of hospital Stay; WT: weight at term (weight at 37 weeks’ postnatal age); NRM; NEC-related mortality.

Among the included trials, 13 studies^39–41,44,45,47–53,55^ were judged to be at low risk of bias for allocation concealment. Most studies properly blinded patients and care providers; however, 13 studies had issues in blinding outcome assessors^38–43,46,47,49,52–55^. Eight trials had issues with incomplete outcome reporting (more than 5% missing participant data)^43,46,49–52,54,55^. Table 2 summarizes the risk of bias assessments.

**Table 2:**
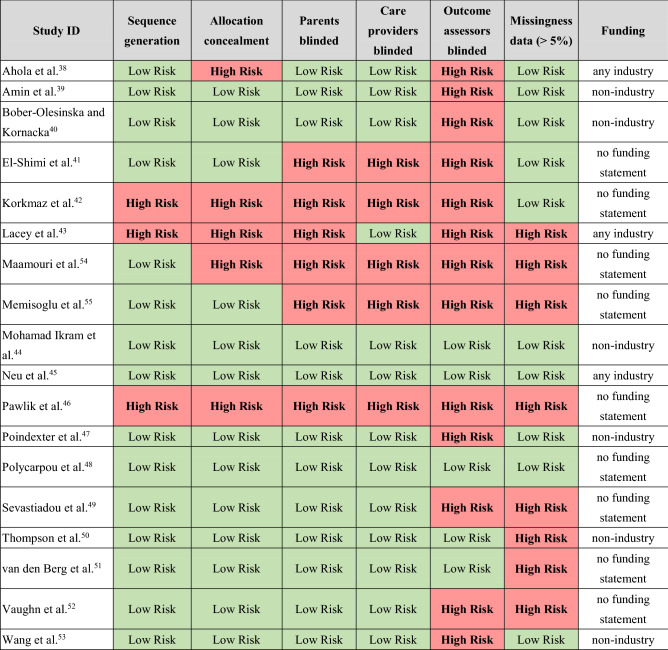
Summary of risk of bias assessments for included studies.

### All-cause mortality

**Figure 3:**
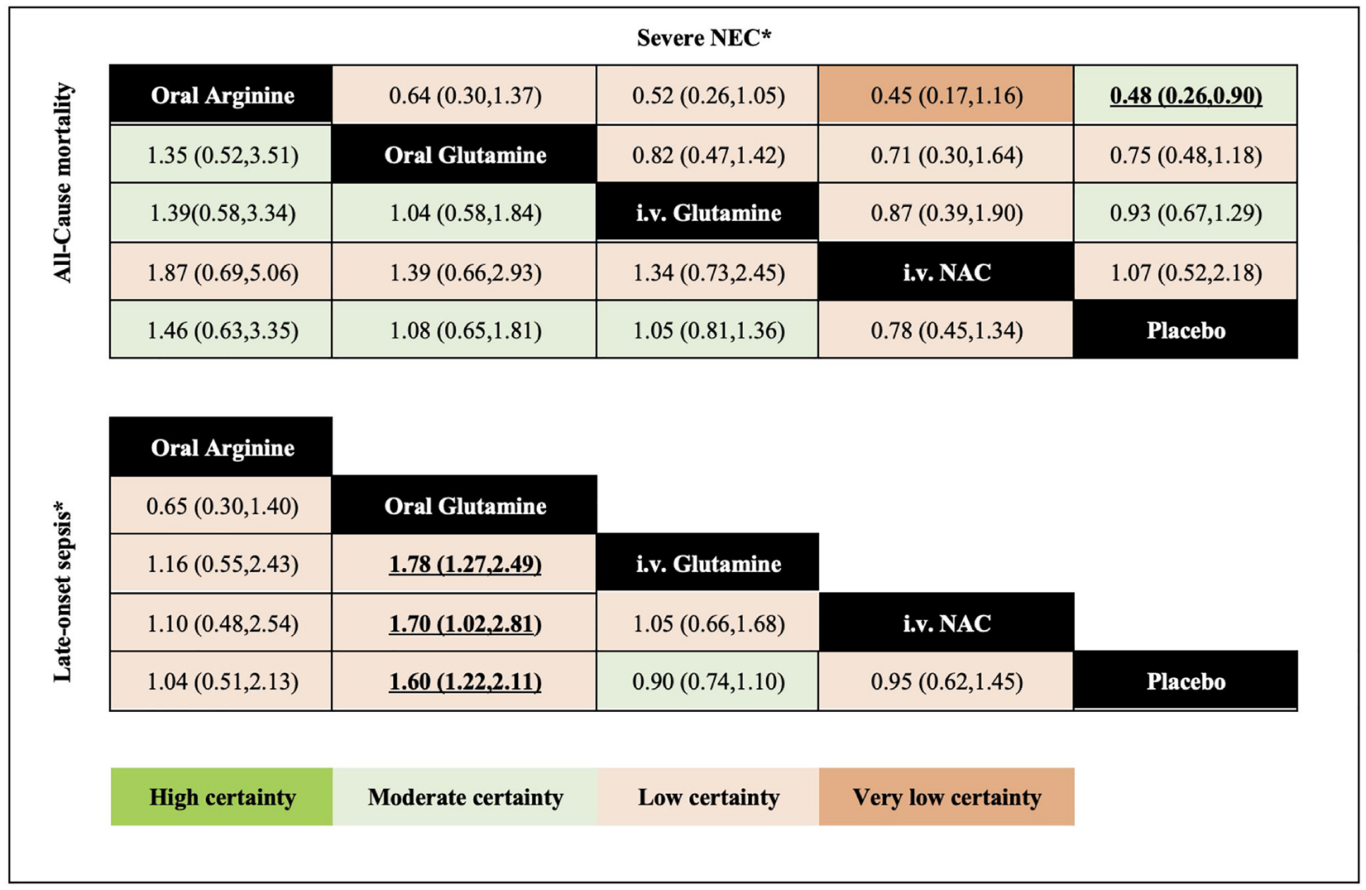
Network meta-analysis results for the primary outcomes. All-cause mortality, severe NEC (stage 2 or more)—top table, and late-onset sepsis—lower table. Results are odds ratio and their corresponding 95% CI. The table should be read from left to right. For all-cause mortality (bottom part of the table), an OR > 1.0 means an increase in mortality. For NEC stage ≥ II (upper part of the table), an OR < 1.0 means a reduction in developing NEC. For culture-proven late-onset sepsis (inferior table), an OR < 1.0 means a reduction in sepsis. Significant results are presented in bold and underlined. The colours represent the GRADE certainty of evidence: green: high; light green: moderate; light orange: low; and orange: very low. *For NEC and sepsis, results are from the fixed-effect model. *i.v.* intravenous, *NAC* N-acetyl cysteine, *NEC* necrotizing enterocolitis.

**Figure 4:**
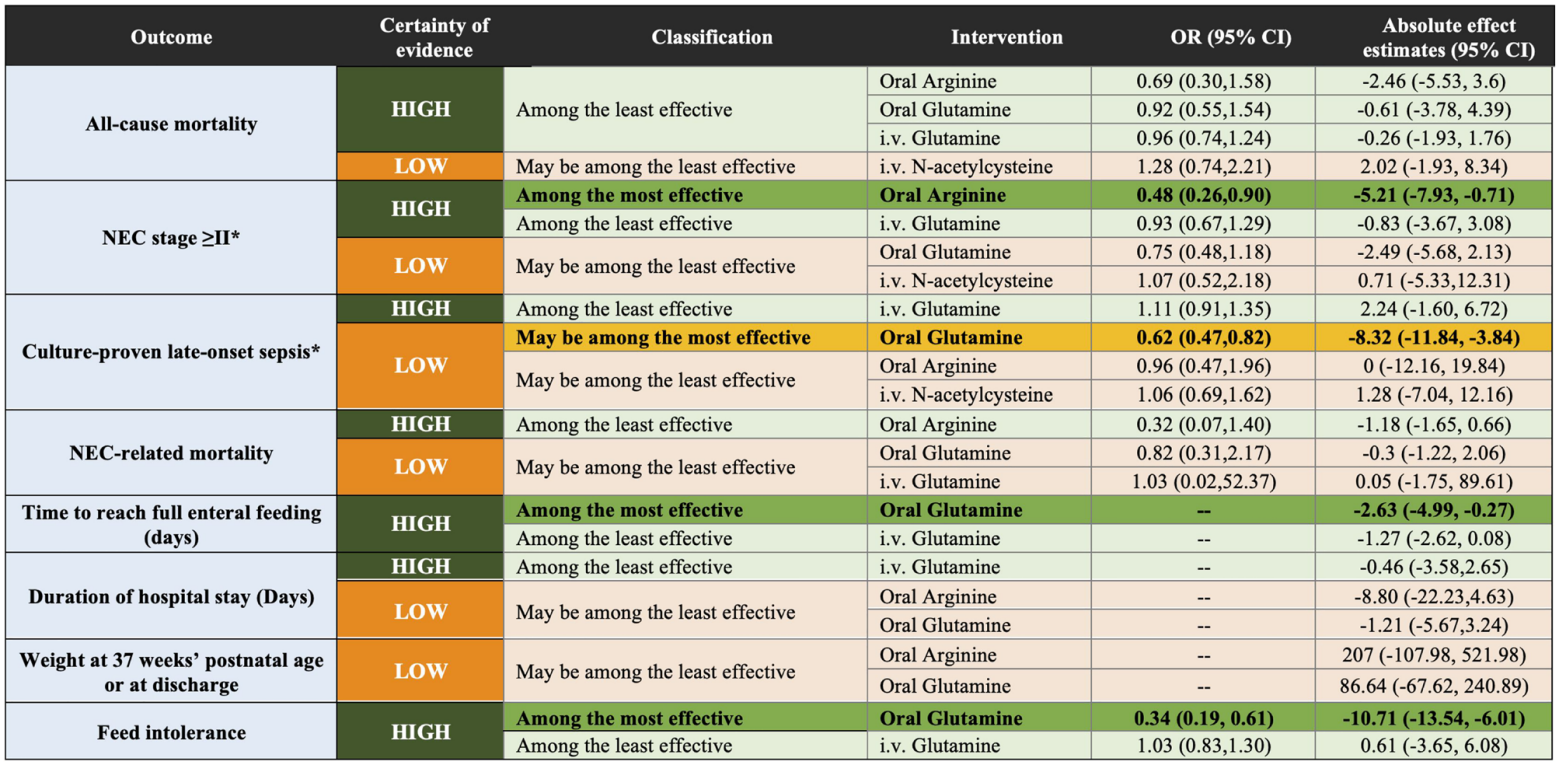
Network meta-analysis summary results sorted based on GRADE certainty of evidence and treatment effects for the comparisons of active treatments versus placebo for all outcomes. NMA results are sorted based on the GRADE certainty of the evidence for comparing active treatments versus placebo for all outcomes. (see “Summary and Certainty of the Results” subsection in the Methods section for more details about the categories). In the second column, green cells denote high certainty of the evidence, and orange cells denote low certainty of the evidence. In columns three to six, dark green denotes that with high certainty the intervention is among the most effective; light green denotes that with high certainty the intervention is among the least effective; dark orange denotes that with low certainty the intervention is among the most effective; and light orange denotes that with low certainty the intervention is among the least effective. We present the relative and absolute effect estimates for all-cause mortality, NEC ≥ stage II, culture-proven late-onset sepsis, NEC-related mortality, and feed intolerance. For continuous outcomes (Time to reach full enteral feed in days and duration of the hospital stay), results are the mean difference (absolute effect). In bold, interventions that showed statistical significance in comparison to placebo. *95% CI* 95% confidence interval, *GRADE* Grading of Recommendations, Assessment, Development, and Evaluation, *i.v.* intravenous, *NEC* necrotizing enterocolitis. *For NEC and sepsis, results are from fixed-effect model.

The 14 studies reporting mortality enrolled 3407 infants and informed five direct comparisons for 3 of which there were 2 or more studies available for conventional pairwise meta-analysis (Supplementary table 2). We did not observe any evidence of global or loop-specific incoherence in this network (Supplementary table 15). The results of NMA did not show any statistically significant benefit for any of the amino acids compared to placebo (low to moderate certainty—Figs. 3 and 4).

### Severe NEC

The 18 studies reporting severe NEC (Bell’s stage II or more) enrolled 3702 infants and informed five direct comparisons for 3 of which there were two or more studies available for conventional pairwise meta-analysis (Supplementary table 3). We did not observe any evidence of global or loop-specific incoherence in this network (Supplementary table 16).

The results of random-effect model NMA was associated with spuriously wide confidence interval (i.e., 95% CI of the NMA estimates were wider than those from the direct or the indirect estimates), and therefore we decided to report results of the fixed-effect model for this outcome. Moderate certainty evidence suggested that oral arginine may reduce the likelihood of developing severe NEC in preterm infants compared to placebo (OR 0.48, 95% CI 0.26–0.90; moderate certainty—absolute risk reduction [ARR]: − 5.21% [95% CI − 7.93% to − 0.71%]). The NMA results showed no other amino acids has statistically significant benefit compared to placebo (Figs. 3 and 4). The network estimates from random-effects model are provided in Supplementary table 8.

### Culture proven late-onset sepsis

The 15 studies reporting culture proven LOS enrolled 3217 infants and informed four direct comparisons from the pairwise meta-analyses (Supplementary table 4). We did not observe any evidence of incoherence in this network.

The results of random-effect model NMA was associated with spuriously wide confidence interval, and therefore we decided to report results of the fixed-effect model for this outcome. Oral glutamine demonstrated statistically significant reduction in likelihood of LOS compared to placebo (OR 0.62; 95% CI 0.47, 0.82; low certainty—ARR = − 8.32%, 95% CI − 11.84% to − 3.84%) (Figs. 3 and 4). The network estimates from random-effects model are provided in Supplementary table 9.

### Other patient-important outcomes

Six studies (1219 infants) reported the NEC-related mortality. Of the four available direct comparisons, two had two or more studies. None of the amino acids showed statistically significant benefit compared to placebo (Fig. 4 and Supplementary table 10).

Time to reach full enteral feeding was reported in seven studies (1,858 infants) informing two direct comparisons, both comparisons had two or more studies. Oral glutamine reduced mean number of days to reach full enteral feeding by 2.63 days compared with placebo (95% CI − 4.99 to − 0.27; moderate certainty) (Supplementary table 11).

The 11 studies reporting duration of hospital stay enrolled 2479 infants and informed 3 direct comparisons of which 2 had 2 or more studies. None of the amino acids showed statistically significant benefit compared to placebo (Fig. 4 and Supplementary table 12).

Networks of length of hospitalization (days) and time to reach full enteral feeding were sparse and there was no global or loop-specific incoherence. We did not observe any evidence of global or loop-specific incoherence in the network of NEC-related mortality (Supplementary tables 17).

Only three studies (2184 infants) reported feeding intolerance. We did not perform NMA for this outcome. The results of conventional meta-analysis showed a reduction in the likelihood of feed intolerance for oral glutamine compared with placebo (OR 0.34; 95% CI 0.19, 0.61; I^2^ = 0%; moderate certainty—ARR = − 10.71%; 95% CI − 13.54% to − 6.01%) (Supplementary table 13).

The weight at 37 weeks’ postnatal age or at discharge was reported in three studies (770 infants) with two trials comparing oral glutamine to placebo and one trial comparing oral arginine to placebo. Conventional meta-analysis showed no statistically significant benefit for oral glutamine compared to placebo (Supplementary table 14).

Few trials reported on adverse events. Polycarpou et al.^48^ declared that no adverse effects were observed in neonates receiving oral l-arginine, and Amin et al.^39^ and El-Shimi et al.^41^ reported no increase in incidence of hypotension or hyperglycemia among infants supplemented with oral arginine. Ahola et al.^38^ reported no adverse effects related to i.v. N-acetylcysteine.

### Additional analyses

Results of the meta-regression analyses adjusted by mean birth weight, mean gestational age and the proportion of infants delivered by C-section are presented in Supplementary tables 5, 6 and 7. We found a significant effect modification for the comparison of oral glutamine versus placebo for severe NEC and LOS when adjusting for birth weight and gestational age showing more benefit for infants with higher birth weight and older gestational age (Supplementary tables 6 and 7). Supplementary table 18 provides SUCRA values and mean ranks for different amino acids, and Supplemental figures 1 to 6 provide ranking probabilities. For all-cause mortality and severe NEC, SUCRA suggested that oral arginine may be the best intervention, but the mean rank was not remarkably different from oral glutamine and i.v. glutamine. For LOS, oral glutamine showed the highest SUCRA, and the mean rank concurs with this finding (mean rank = 1.1).

## Discussion

### Main findings

In this systematic review and NMA, we summarized evidence from 18 studies (3702 infants) and found that three different amino acids have been studied to prevent mortality and morbidity in preterm infants. Low to moderate certainty evidence indicated no meaningful impact on all-cause mortality of amino acids relative to placebo (Fig. 2). Low certainty evidence indicated that oral arginine may prevent NEC stage II and III compared with placebo and low certainty evidence suggested that oral glutamine prevented culture-proven sepsis compared with placebo. No evidence supports the preventive effects of other comparisons on NEC ≥ II and culture-proven sepsis. Moderate certainty evidence shows that the number of days to reach full enteral feed was reduced by 2.63 days using oral glutamine relative to placebo; however, it may not include a clinically meaningful reduction. Low certainty evidence of no impact was found for oral arginine or oral glutamine for weight at 37 weeks postnatal age at discharge versus placebo.

We did not find any differences between arginine, glutamine (oral or i.v.) and NAC in all-cause mortality and severe NEC. Nonetheless, with low certainty, we found that oral glutamine may be superior not only to placebo but also to the other amino acids in reducing the incidence of LOS.

### Strengths and limitations

Our review has several strengths. This is the most comprehensive systematic review on this topic to date, using a broad search to identify and include all available literature without language restrictions. We applied state-of-the-art methodologies for developing NMA, according to a prespecified protocol and statistical-analysis plan, and we followed the PRISMA-NMA guidance^18^. Moreover, we used the GRADE approach^36,37^ to assess the certainty of the NMA effect estimates and to draw conclusions applying the most recent methodological approach, which considers the effect and the certainty of the evidence.

Our review is not free of limitations. A low number of studies relative to the number of comparisons considered, and the scarce direct evidence comparing the amino acids among them resulted in mostly indirect comparisons and low confidence in estimates for many key analyses. Only one trial directly compared two active treatments (oral arginine versus oral glutamine), while the rest of the studies compared one active treatment to placebo (Table 1). Due to this scarcity of direct evidence among active interventions, we were unable to further explore the effect of variability in supplementation details, including dosages administered and durations of the intervention.

### Comparison with other studies

Two Cochrane reviews have addressed the effectiveness of amino acids for preterm infants. Moe-Byrne et al.^15^ included 12 trials and concluded that glutamine did not have an effect on mortality or the incidence of invasive infection or NEC compared to control. Our main findings differ from theirs for two reasons. First, although they performed subgroup analyses by administration route, they drew their conclusions from the syntheses of both oral and i.v. glutamine. Instead, we considered oral and i.v. glutamine as different interventions, and thus, we created one node for each and could find that oral administration may be useful for preventing LOS (low certainty). Lastly, NMA provides effect estimates of interventions not only compared to placebo or no treatment but also among all the interventions. Therefore, we could also describe how oral glutamine was significantly superior to i.v. glutamine.

The reason for this difference according to the administration route may be related to intestinal permeability. As the most abundant free amino acid in the human body, glutamine helps to maintain gut barrier integrity by promoting enterocyte proliferation, regulating tight junction proteins, suppressing pro-inflammatory signaling pathways, and protecting cells against apoptosis and cellular stresses. Glutamine supplementation could balance its depletion during sepsis and improve clinical outcomes^56,57^. In fact, Sevastiadou et al.^49^ found a significant reduction of intestinal permeability with oral glutamine. This reduction might be associated with less bacterial translocation and therefore, less intestinal-related sepsis. Although we cannot be sure about the effect of oral glutamine, our results warrant further trials comparing it with i.v. glutamine and other interventions.

In 2017, the review by Shah et al.^14^ included three trials and reported a positive effect of arginine supplementation in reducing the risk of development of NEC stage I, but not for NEC stages II or III. In our NMA, we found a potential reduction of NEC stage ≥ II when supplementing with oral arginine. However, these findings should be interpreted with caution because of the small number of included studies and the potential of biases in the included trials, which explain the low certainty of the evidence. Similar to glutamine, arginine may have a potential beneficial effect on the immature intestine, which could explain its effect on reducing sepsis occurrence^58^.

Although some of our findings agree with previous reviews, we present some novel results. First, our review is the first to compare all the available amino acids simultaneously, and thus, we can provide effect estimates of comparisons among them. For instance, our league tables (Fig. 2) show how oral glutamine is not only more effective than placebo but also more effective than i.v. glutamine. Second, we applied GRADE methodology to assess the certainty of the evidence so readers can identify what comparisons benefit from further research more than others. Third, we provide updated evidence from studies that had not been synthesized before and included three new studies in addition to the total 15 studies from the previous two Cochrane reviews, and as such, this work will be useful to inform decision-making and for updating guidelines.

In summary, oral glutamine and arginine seem to reduce culture-proven late-onset sepsis and NEC ≥ II, respectively. These findings need to be confirmed in future large trials. We encourage trialists to design large high-quality RCTs directly comparing oral arginine, oral glutamine, and placebo to confirm these effects.

## Conclusion

The evidence from RCTs suggests that amino acid supplementation, including glutamine, arginine and N-acetylcysteine, does not have an important effect on mortality in preterm infants. However, in comparison to the placebo, oral glutamine has shown to be superior for reducing the days to reach full enteral feeding (moderate certainty), it may be superior for preventing the culture-proven late-onset sepsis (low certainty), and oral arginine may be effective for reducing NEC stage ≥ II (moderate certainty). Lastly, i.v. glutamine and NAC seem to be similar to placebo for all the measured outcomes. These findings should be interpreted with caution because of the small number of studies included and the potential risk of biases.

## Supplementary Information

Supplementary Information.

## Data Availability

All data generated or analyzed during this study are included in this published article and its supplementary information files.
