# Supplementary material for: Amino acids for the prevention of mortality and morbidity in preterm infants: a systematic review and network meta-analysis
Source: Sci Rep. 2022 Oct 31;12:18333. doi: 10.1038/s41598-022-21318-w (PMC9622873; doi:10.1038/s41598-022-21318-w)
Supplement: Supplementary file 1 — Supplementary Information. [file 41598_2022_21318_MOESM1_ESM.docx]

**Supplementary Information**

**Amino acids for the prevention of mortality and morbidity in preterm infants: A systematic review and network meta-analysis**

Xiaoqin Wang, Behnam Sadeghirad, Rebecca Morgan, Dena Zeratkaar, Yaping Chang, Holly Crandon, Rachel Couban, Farid Foroutan, Ivan D. Florez

**Content**

[Supplementary table 1. Search Strategy of different databases 2](#_Toc74755872)

[Supplementary table 2. Direct, indirect and NMA estimates for all-cause mortality 4](#_Toc74755873)

[Supplementary table 3. Direct, indirect and NMA estimates NEC stage II 5](#_Toc74755874)

[Supplementary table 4. Direct, indirect and NMA estimates culture proven late-onset sepsis 6](#_Toc74755875)

[Supplementary table 5. Meta-regression analyses for all-cause mortality 7](#_Toc74755876)

[Supplementary table 6. Meta-regression analyses for NEC stage II 7](#_Toc74755877)

[Supplementary table 7. Meta-regression analyses for culture-proven late-onset sepsis 7](#_Toc74755878)

[Supplementary table 8. Sensitivity analysis for NEC II with random effects model 9](#_Toc74755879)

[Supplementary table 9. Sensitivity analysis for culture proven late-onset sepsis with random effects model 10](#_Toc74755880)

[Supplementary table 10. Direct, indirect and NMA estimates for NEC-related mortality 11](#_Toc74755881)

[Supplementary table 11. Direct, indirect and NMA estimates for time to reach full enteral feeding (days) 12](#_Toc74755882)

[Supplementary table 12. Direct, indirect and NMA estimates for duration of hospital stay (days) 12](#_Toc74755883)

[Supplementary table 13. Direct estimates for feeding intolerance 12](#_Toc74755884)

[Supplementary table 14. Direct estimates for weight at 37 weeks’ postnatal age or at discharge 13](#_Toc74755885)

[Supplementary table 15. Incoherence analysis by the node-splitting model of all-cause mortality 14](#_Toc74755886)

[Supplementary table 16. Inconsistency analysis by the node-splitting model of NEC stage II 14](#_Toc74755887)

[Supplementary table 17. Inconsistency analysis by the node-splitting model of NEC-related mortality 14](#_Toc74755888)

[Supplementary table 18. Cumulative ranking of interventions for different outcomes 15](#_Toc74755889)

[Supplementary figure 1. Plots of the SUCRAs for all-cause mortality 16](#_Toc74755890)

[Supplementary figure 2. Plots of the SUCRAs for NEC stage II 16](#_Toc74755891)

[Supplementary figure 3. Plots of the SUCRAs for culture-proven Late-onset Sepsis 17](#_Toc74755892)

[Supplementary figure 4. Plots of the SUCRAs for NEC-related mortality 17](#_Toc74755893)

[Supplementary figure 5. Plots of the SUCRAs for time to achieve full enteral feeding (days) 18](#_Toc74755894)

[Supplementary figure 6. Plots of the SUCRAs for duration of hospitalization (days) 18](#_Toc74755895)

# Supplementary table 1. Search Strategy of different databases

| **OVID Medline**  1 Enterocolitis, Necrotizing/  2 (necroti?ing enterocolitis or enterocolitis necroticans or nec or typhlitis or pneumatosis intestinalis).tw.  3 1 or 2  4 randomized controlled trial.pt.  5 controlled clinical trial.pt.  6 randomized.ab.  7 placebo.ab.  8 drug therapy.fs.  9 randomly.ab.  10 trial.ab.  11 groups.ab.  12 or/4-11  13 exp animals/ not humans.sh.  14 12 not 13  15 3 and 14 |
| --- |
| **Embase**  1 necrotizing enterocolitis/  2 (necroti?ing enterocolitis or enterocolitis necroticans or nec or typhlitis or  pneumatosis intestinalis).tw.  3 1 or 2  4 crossover-procedure/  5 double-blind procedure/  6 randomized controlled trial/  7 single-blind procedure/  8 ((doubl* adj blind*) or (singl* adj blind*) or allocat* or assign* or cross  over* or crossover* or factorial* or placebo* or random* or volunteer*).tw.  9 or/4-8  10 3 and 9  11 Animal experiment/ not (human experiment/ or human/)  12 10 not 11  13 limit 12 to em=201848-201952  14 limit 12 to yr="2019 -Current"  15 13 or 14 |
| **Central**  #1 MeSH descriptor: [Enterocolitis, Necrotizing] explode all trees  #2 enterocolitis and (necrot*)  #3 nec  #4 pneumatosis intestinalis  #5 (necroti?ing enterocolitis or enterocolitis necroticans or nec or typhlitis or pneumatosis intestinalis)  #6 typhlitis  #7 #1 or #2 or #3 or #4 or #5 or #6 with Publication Year from 2019 to 2019, in Trials |
| **Web of Science**  # 1 TS=("necrotising enterocolitis" or "necrotizing enterocolitis" or "enterocolitis necroticans" or "nec" or "typhlitis" or "pneumatosis intestinalis")  # 2 TS=((doubl* NEAR blind*) or (singl* NEAR blind*) or allocat* or assign* or "cross over*" or crossover* or factorial* or placebo* or random* or volunteer*)  # 3 #2 AND #1  # 4 TS=(rat or rats or mouse or mice or pig or pigs or piglet or piglets or porcine)  # 5 #3 not #4  CINAHL  S1 (MH "Enterocolitis, Necrotizing")  S2 TX "necrotising enterocolitis" or "necrotizing enterocolitis" or "enterocolitis necroticans" or "nec" or "typhlitis" or "pneumatosis intestinalis"  S3 S1 OR S2  S4 (MH "Clinical Trials+")  S5 "randomi?ed controlled trial*"  S6 clinical W3 trial  S7 "double-blind"  S8 "single-blind"  S9 "triple-blind"  S10 S4 OR S5 OR S6 OR S7 OR S8 OR S9  S11 (MH "Placebo Effect")  S12 (MH "Placebos")  S13 "placebo*"  S14 "random*"  S15 S11 OR S12 OR S13 OR S14  S16 (MH "Random Sample+")  S17 (MH "Study Design") OR (MH "Crossover Design") OR (MH "Experimental Studies+")  S18 "latin square"  S19 (MH "Comparative Studies")  S20 (MH "Evaluation Research+")  S21 (MH "Prospective Studies+")  S22 S16 OR S17 OR S18 OR S19 OR S20 OR S21  S23 S10 OR S15 OR S22  S24 S3 AND S23 |
| **ProQuest Dissertations & Theses A&I**  TI,AB,FT("necrotising enterocolitis" OR "necrotizing enterocolitis" OR "enterocolitis necroticans" OR "typhlitis" OR "pneumatosis intestinalis") AND TI,AB,FT((doubl* NEAR/1 blind*) OR (singl* NEAR/1 blind*) OR allocat* OR assign* OR "cross over*" OR crossover* OR factorial* OR placebo* OR random* OR volunteer*) NOT TI,AB,FT(rat OR rats OR mouse OR mice OR pig OR pigs OR piglet OR piglets OR porcine OR animal)Limits applied |

# Supplementary table 2. Direct, indirect and NMA estimates for all-cause mortality

| **Comparison** | **Number of trials with direct evidence, n** | **Direct estimates***  **OR (95% CI); CoE** | **Indirect estimates***  **OR (95% CI); CoE** | **NMA estimates** | | |
| --- | --- | --- | --- | --- | --- | --- |
|  |  |  |  | **OR (95% CI)** | **%RD (95%CI)** | **CoE** |
| IV Glutamine vs IV NAC | 0 | - | 0.75 (0.41,1.36); Moderate^a^ | 0.75 (0.41,1.36) | - | Low^b^ |
| IV Glutamine vs Oral Arginine | 0 | - | 1.41 (0.59,3.37); High | 1.39 (0.58,3.34) | - | Moderate^b^ |
| IV Glutamine vs Oral Glutamine | 0 | - | 1.39 (0.58,3.34); High | 1.04 (0.58,1.84) | - | Moderate^b^ |
| IV Glutamine vs placebo | 5 | 0.96 (0.74, 1.24); High | - | 0.96 (0.74, 1.24) | -0.26 (-1.93, 1.76) | Moderate^b^ |
| IV NAC vs Oral Arginine | 0 | - | 1.87 (0.69,5.06); Moderate^a^ | 1.87 (0.69,5.06) | - | Low^b^ |
| IV NAC vs Oral Glutamine | 0 | - | 1.39 (0.66,2.93); Moderate^a^ | 1.39 (0.66,2.93) | - | Low^b^ |
| IV NAC vs placebo | 1 | 1.28 (0.74,2.21); Moderate^a^ | - | 1.28 (0.74,2.21) | 2.02 (-1.93, 8.34) | Low^b^ |
| Oral Arginine vs Oral Glutamine | 1 | 1 (0.13, 7.72); Moderate^a^ | 0.68 (0.23,2.02); High | 0.74 (0.28,1.94) | - | Moderate^b^ |
| Oral Arginine vs placebo | 3 | 0.72 (0.19, 2.71); High | 5.44 (0.17, 174.93); Moderate^a^ | 0.69 (0.30,1.58) | -2.46 (-5.53, 3.6) | Moderate^b^ |
| Oral Glutamine vs placebo | 6 | 0.90 (0.49,1.66); High | 4.21 (0.09, 190.76); Moderate^a^ | 0.92 (0.55,1.54) | -0.61 (-3.78, 4.39) | Moderate^b^ |

Note: OR, odds ratio; CoE, certainty of evidence; NAC, N-acetylcysteine RD, risk difference.

* Direct and indirect estimates were judged following the GRADE approach for NMA. As such, Imprecision criterion was not applied to them, but to the final NMA estimates

a Rated down for risk of bias

b Rated down for imprecision

# Supplementary table 3. Direct, indirect and NMA estimates NEC stage II

| **Comparison** | **Number of trials with direct evidence, n** | **Direct estimates**  **OR (95% CI); CoE** | **Indirect estimates**  **OR (95% CI); CoE** | **NMA estimates** | | |
| --- | --- | --- | --- | --- | --- | --- |
|  |  |  |  | **OR (95% CI): COE** | **%RD (95%CI)** | **CoE** |
| IV Glutamine vs IV NAC | 0 | - | 0.87 (0.39,1.90); Moderate ^a^ | 0.87 (0.39,1.90) | - | Low^c^ |
| IV Glutamine vs Oral Arginine | 0 | - | 1.93 (0.95,3.91); Moderate ^b^ | 1.93 (0.95,3.91) | - | Low^c^ |
| IV Glutamine vs Oral Glutamine | 0 | - | 1.23 (0.70,2.13); Moderate ^b^ | 1.23 (0.70,2.13) | - | Low^c^ |
| IV Glutamine vs placebo | 5 | 0.93 (0.67, 1.29); High | - | 0.93 (0.67,1.29) | -0.83 (-3.67, 3.08) | Moderate^c^ |
| IV NAC vs Oral Arginine | 0 | - | 2.23 (0.86,5.75); Moderate ^a^ | 2.23 (0.86,5.75) | - | Very Low^d^ |
| IV NAC vs Oral Glutamine | 0 | - | 1.42 (0.61,3.28); Moderate ^a^ | 1.42 (0.61,3.28) | - | Low^c^ |
| IV NAC vs placebo | 1 | 1.07 (0.52,2.18); Moderate ^a^ | - | 1.07 (0.52,2.18) | 0.71 (-5.33,12.31) | Low^c^ |
| Oral Arginine vs Oral Glutamine | 1 | 5.43 (0.25, 118.96); Moderate ^a^ | 0.78 (0.24,2.48); Moderate ^b^ | 0.64 (0.30,1.37) | - | Low^c^ |
| Oral Arginine vs placebo | 4 | 0.46 (0.25, 0.87); Moderate ^a^ | 30.48 (0.07,1.40E+04); Moderate ^a^ | **0.48 (0.26,0.90)** | **-5.21 (-7.93, -0.71)** | Moderate |
| Oral Glutamine vs placebo | 7 | 0.43 (0.18, 1.06); Moderate^b^ | 0.45 (0.01,27.91); Moderate ^a^ | 0.75 (0.48,1.18) | -2.49 (-5.68, 2.13) | Low^c^ |

Note: OR, odds ratio; CoE, certainty of evidence; NAC, N-acetylcysteine RD, risk difference. The results are from fixed-effect model

a Rated down for risk of bias

b Rated down for heterogeneity (I^2^=57.8%; P=0.027)

c Rated down for imprecision

d Rated down two levels for imprecision

# Supplementary table 4. Direct, indirect and NMA estimates culture proven late-onset sepsis

| **Comparison** | **Number of trials with direct evidence, n** | **Direct estimates***  **OR (95% CI); CoE** | **Indirect estimates***  **OR (95% CI); CoE** | **NMA estimates** | | |
| --- | --- | --- | --- | --- | --- | --- |
|  |  |  |  | **OR (95% CI)** | **%RD (95%CI)** | **CoE** |
| IV Glutamine vs IV NAC | 0 | - | 0.92 (0.34,2.51); Moderate^a^ | 1.05 (0.66,1.68) | - | Low^c^ |
| IV Glutamine vs Oral Arginine | 0 | - | 1.16 (0.55,2.43); Moderate^a^ | 1.16 (0.55,2.43) | - | Low^c^ |
| IV Glutamine vs Oral Glutamine | 0 | - | 1.78 (1.27,2.49); Low ^a,b^ | **1.78 (1.27,2.49)** | - | Low^c^ |
| IV Glutamine vs placebo | 5 | 1.11(0.91, 1.35); High | - | 1.11 (0.91,1.35) | 2.24 (-1.60, 6.72) | Moderate^c^ |
| IV NAC vs Oral Arginine | 0 | - | 1.09 (0.32,3.78); Moderate^a^ | 1.10 (0.48,2.54) | - | Low^c^ |
| IV NAC vs Oral Glutamine | 0 | - | 2.47 (0.88,6.94); Moderate ^a^ | **1.70 (1.02,2.81)** | - | Low^c^ |
| IV NAC vs placebo | 1 | 1.06 (0.69,1.62); Moderate^a^ | - | 1.06 (0.69,1.62) | 1.28 (-7.04, 12.16) | Low^c^ |
| Oral Arginine vs Oral Glutamine | 0 | - | 2.26 (0.78,6.52); Moderate ^a^ | 1.54 (0.71,3.30) | - | Low^c^ |
| Oral Arginine vs placebo | 2 | 0.96 (0.47,1.96); Moderate^a^ | - | 0.96 (0.47,1.96) | 0 (-12.16, 19.84) | Low^c^ |
| Oral Glutamine vs placebo | 7 | 0.39 (0.21,0.72); Low^a,b^ | - | **0.62 (0.47,0.82)** | **-8.32 (-11.84, -3.84)** | Low |

Note: OR, odds ratio; CoE, certainty of evidence; NAC, N-acetylcysteine RD, risk difference. The results are from fixed-effect model

a Rated down for risk of bias

b Rated down for inconsistency (I^2^=59.5%, P=0.022)

c Rated down for imprecision

# Supplementary table 5. Meta-regression analyses for all-cause mortality

| **Unadjusted** | **Compared to placebo** | **OR** | **95% CIs** | **P value** |
| --- | --- | --- | --- | --- |
| Heterogeneity  (Tau<0.001) | IV Glutamine | 0.96 | (0.74, 1.24) | 0.736 |
|  | NAC | 1.28 | (0.74,2.21) | 0.371 |
|  | Oral Arginine | 0.69 | (0.30,1.58) | 0.376 |
|  | Oral Glutamine | 0.92 | (0.55,1.54) | 0.762 |
| **Adjusted for** | **Compared to placebo** | **OR** | **95%CIs** | **P value** |
| Birth weight (100g) | IV glutamine | 0.97 | (0.87,1.08) | 0.555 |
|  | IV NAC * | - | - | - |
|  | Oral arginine | 0.62 | (0.31,1.26) | 0.188 |
|  | Oral glutamine | 0.92 | (0.65,1.31) | 0.661 |
| Gestational age(week) | IV glutamine | 1.11 | (0.54,2.30) | 0.776 |
|  | IV NAC * | - | - | - |
|  | Oral arginine | 0.60 | (0.27,1.34) | 0.212 |
|  | Oral glutamine | 0.90 | (0.58,1.38) | 0.62 |
| % infants delivered by caesarean section | IV glutamine | 1.00 | (0.89,1.11) | 0.931 |
|  | IV NAC * | - | - | - |
|  | Oral arginine | 0.93 | (0.75,1.14) | 0.486 |
|  | Oral glutamine | 0.96 | (0.91,1.01) | 0.102 |

*Only one study compared IV NAC vs placebo; NAC, N-acetylcysteine

# Supplementary table 6. Meta-regression analyses for NEC stage II

| **Unadjusted** | **Compared to placebo** | **OR** | **95% CIs** | **P value** |
| --- | --- | --- | --- | --- |
| Heterogeneity (Tau=0.450) | IV Glutamine | 0.76 | (0.36, 1.61) | 0.475 |
|  | NAC | 1.07 | (0.34, 3.32) | 0.909 |
|  | Oral Arginine | 0.49 | (0.23, 1.05) | 0.068 |
|  | Oral Glutamine | 0.55 | (0.25, 1.19) | 0.129 |
| **Adjusted for** | **Compared to placebo** | **OR** | **95% CIs** | **P value** |
| Birth weight (100g) | IV glutamine | 0.95 | (0.88,1.04) | 0.297 |
|  | IV NAC* | - | - | - |
|  | Oral arginine | 1.00 | (0.65,1.52) | 0.989 |
|  | Oral glutamine | 0.59 | (0.43,0.81) | **0.001** |
| Gestational age (week) | IV glutamine | 0.39 | (0.10,1.47) | 0.163 |
|  | IV NAC | - | - | - |
|  | Oral arginine | 1.12 | (0.7,1.80) | 0.631 |
|  | Oral glutamine | **0.54** | **(0.37,0.80)** | **0.002** |
| % infants delivered by caesarean section | IV glutamine | 0.98 | (0.85,1.13) | 0.789 |
|  | IV NAC | - | - | - |
|  | Oral arginine | 0.99 | (0.7,1.38) | 0.932 |
|  | Oral glutamine | 0.96 | (0.89,1.05) | 0.377 |

*Only one study compared IV NAC vs placebo; NAC, N-acetylcysteine

# Supplementary table 7. Meta-regression analyses for culture-proven late-onset sepsis

| **Unadjusted** | **Compared to placebo** | **OR** | **95% CIs** | **P value** |
| --- | --- | --- | --- | --- |
| Heterogenity  (Tau=0.366) | IV Glutamine | 0.98 | (0.60, 1.61) | 0.931 |
|  | NAC | 1.06 | (0.46, 2.43) | 0.896 |
|  | Oral Arginine | 0.97 | (0.40, 2.32) | 0.938 |
|  | Oral Glutamine | ***0.44*** | ***(0.26, 0.74)*** | 0.002 |
| **Adjusted for** | **Compared to placebo** | **OR** | **95% CIs** | **P value** |
| Birth weight (100g) | IV glutamine vs placebo | 0.98 | (0.92,1.03) | 0.479 |
|  | IV NAC vs placebo* | - | - | - |
|  | Oral arginine vs placebo | 1.36 | (0.40,4.58) | 0.618 |
|  | Oral glutamine vs placebo | **0.73** | **(0.61,0.87**) | **0.001** |
| Gestational age (week) | IV glutamine vs placebo | 0.74 | (0.44,1.23) | 0.242 |
|  | IV NAC vs placebo | - | - | - |
|  | Oral arginine vs placebo | 1.18 | (0.61,2.28) | 0.618 |
|  | Oral glutamine vs placebo | **0.67** | **(0.53,0.85)** | **0.001** |
| **% infants delivered by caesarean section** | Not enough data for this variable | | | |

*Only one study compared IV NAC vs placebo; NAC, N-acetylcysteine

# Supplementary table 8. Sensitivity analysis for NEC II with random effects model

| **Comparison** | **Number of trials with direct evidence, n** | **Direct estimates**  **OR (95% CI); CoE** | **Indirect estimates**  **OR (95% CI); CoE** | **NMA estimates** | | |
| --- | --- | --- | --- | --- | --- | --- |
|  |  |  |  | **OR (95% CI): COE** | **%RD (95%CI)** | **CoE** |
| IV Glutamine vs IV NAC | 0 | - | 0.71 (0.18,2.78); Moderate^a^ | 0.71 (0.18,2.78) | - | Low^c^ |
| IV Glutamine vs Oral Arginine | 0 | - | 1.56 (0.53,4.61); Moderate^a^ | 1.56 (0.53,4.61) | - | Low^c^ |
| IV Glutamine vs Oral Glutamine | 0 | - | 1.38 (0.52,3.70); Moderate^b^ | 1.38 (0.52,3.70) | - | Low^c^ |
| IV Glutamine vs placebo | 5 | 0.93 (0.67, 1.29); High | - | 0.76 (0.36,1.61) | -2.37 (-6.99, 6.39) | Moderate^c^ |
| IV NAC vs Oral Arginine | 0 | - | 2.19 (0.56,8.64); Moderate^a^ | 2.19 (0.56,8.64) | - | Very Low^d^ |
| IV NAC vs Oral Glutamine | 0 | - | 1.95 (0.49,7.69); Moderate^a^ | 1.95 (0.49,7.69) | - | Very Low^d^ |
| IV NAC vs placebo | 1 | 1.07 (0.52,2.18); Moderate^a^ | - | 1.07 (0.34,3.32) | 0.71 (-7.1, 21.55) | Low^c^ |
| Oral Arginine vs Oral Glutamine | 1 | 5.43 (0.25, 118.96);Moderate^a^ | 0.78 (0.24,2.48); Moderate^b^ | 0.89 (0.30,2.65) | -0.00(-0.10,0.10) | Low^c^ |
| Oral Arginine vs placebo | 4 | 0.46 (0.25, 0.87); Moderate^a^ | 30.48 (0.07,1.40E+04); Moderate^a^ | 0.49 (0.23,1.05) | -5.33 (-8.41, 0.59) | Low^c^ |
| Oral Glutamine vs placebo | 7 | 0.43 (0.18, 1.06); Moderate^b^ | 0.45 (0.01,27.91); Moderate^a^ | 0.55 (0.25,1.19) | -4.5 (-8.17, 2.6) | Low^c^ |

Note: OR, odds ratio; CoE, certainty of evidence; NAC, N-acetylcysteine RD, risk difference.

* Direct and indirect estimates were judged following the GRADE approach for NMA. As such, Imprecision criterion was not applied to them, but to the final NMA estimates

a Rated down for risk of bias

b Rated down for heterogeneity (I^2^=57.8%; P=0.027)

c Rated down for imprecision

d Rated down two levels for imprecision

# Supplementary table 9. Sensitivity analysis for culture proven late-onset sepsis with random effects model

| **Comparison** | **Number of trials with direct evidence, n** | **Direct estimates**  **OR (95% CI); CoE** | **Indirect estimates**  **OR (95% CI); CoE** | **NMA estimates** | | |
| --- | --- | --- | --- | --- | --- | --- |
|  |  |  |  | **OR (95% CI)** | **%RD (95%CI)** | **CoE** |
| IV Glutamine vs IV NAC | 0 | - | 0.92 (0.34,2.51); Moderate^a^ | 0.93 (0.35,2.43) | - | Low^c^ |
| IV Glutamine vs Oral Arginine | 0 | - | 1.16 (0.55,2.43); Moderate^a^ | 1.01 (0.37,2.77) | - | Low^c^ |
| IV Glutamine vs Oral Glutamine | 0 | - | 1.78 (1.27,2.49); Low ^a,b^ | **2.23 (1.11,4.50)** | **-** | Low |
| IV Glutamine vs placebo | 5 | 1.11(0.91, 1.35); High | - | 0.98 (0.60,1.61) | 0 (-8.64, 11.52) | Moderate^c^ |
| IV NAC vs Oral Arginine | 0 | - | 1.09 (0.32,3.78); Moderate^a^ | 1.09 (0.33,3.65) | - | Low^c^ |
| IV NAC vs Oral Glutamine | 0 | - | 2.47 (0.88,6.94); Moderate^a^ | 2.41 (0.91,6.43) | - | Low^c^ |
| IV NAC vs placebo | 1 | 1.06 (0.69,1.62); Moderate^a^ | - | 1.06 (0.46,2.43) | 1.28 (-11.52,22.08) | Low^c^ |
| Oral Arginine vs Oral Glutamine | 0 | - | 2.26 (0.78,6.52); Moderate^a^ | 2.20 (0.79,6.13) | - | Low^c^ |
| Oral Arginine vs placebo | 2 | 0.96 (0.47,1.96); Moderate^a^ | - | 0.97 (0.40,2.32) | -0.32 (-14.08,23.68) | Low^c^ |
| Oral Glutamine vs placebo | 7 | 0.39 (0.21,0.72); Low^a,b^ | - | **0.44 (0.26,0.74)** | **-11.52(-17.28,-3.20)** | Low |

Note: OR, odds ratio; CoE, certainty of evidence; NAC, N-acetylcysteine RD, risk difference.

* Direct and indirect estimates were judged following the GRADE approach for NMA. As such, Imprecision criterion was not applied to them, but to the final NMA estimates

a Rated down for risk of bias

b Rated down for inconsistency (I^2^=59.5%, P=0.022)

c Rated down for imprecision

# Supplementary table 10. Direct, indirect and NMA estimates for NEC-related mortality

| **Comparison** | **Number of trials with direct evidence, n** | **Direct estimates**  **OR (95% CI); CoE** | **Indirect estimates**  **OR (95% CI); CoE** | **NMA estimates** | | |
| --- | --- | --- | --- | --- | --- | --- |
|  |  |  |  | **OR (95% CI)** | **%RD (95%CI)** | **CoE** |
| IV Glutamine vs Oral Arginine | 0 | - | 3.23 (0.05,216.31)Moderate^a^ | 3.23 (0.05,216.31) | - | Very Low^b^ |
| IV Glutamine vs Oral Glutamine | 0 | - | 1.24 (0.02,71.42); Moderate^a^ | 1.24 (0.02,71.42) | - | Very Low^b^ |
| IV Glutamine vs Placebo | 1 | 1.03 (0.02, 52.37); High | - | 1.03 (0.02,52.37) | 0.05 (-1.75, 89.61) | Low^b^ |
| Oral Arginine vs Oral Glutamine | 1 | 3.12 (0.12,80.39); Moderate^a^ | 0.14 (0.01, 1.52); Moderate^a^ | 0.39 (0.07,2.18) | - | Low^c^ |
| Oral Arginine vs Placebo | 3 | 0.27 (0.06, 1.22); High | 37.05 (0.07, 1.90E+04); Moderate^a^ | 0.32 (0.07,1.40) | -1.18 (-1.65, 0.66) | Moderate^c^ |
| Oral Glutamine vs Placebo | 3 | 0.70 (0.18, 2.68);Moderate^a^ | 0.27 (0, 278.55); Moderate^a^ | 0.82 (0.31,2.17) | -0.3 (-1.22, 2.06) | Low^c^ |

Note: OR odds ratio; CoE, certainty of evidence; RD, risk difference.

a Rated down two levels for imprecision

a Rated down for risk of bias

b Rated down two levels for imprecision

c Rated down for imprecision

# Supplementary table 11. Direct, indirect and NMA estimates for time to reach full enteral feeding (days)

| **Comparison** | **Number of trials with direct evidence, n** | **Direct estimates**  **MD (95% CI); CoE** | **Indirect estimates**  **MD (95% CI); CoE** | **NMA estimates**  **MD (95% CI); CoE** |
| --- | --- | --- | --- | --- |
|  |  |  |  |  |
| IV Glutamine vs Oral Glutamine | 0 | - | 1.36 (-1.36,4.08); High | 1.36 (-1.36,4.08); Moderate^b^ |
| IV Glutamine vs placebo | 5 | -1.57 (-3.45, 0.31); High | - | -1.27 (-2.62,0.08); Moderate^b^ |
| Oral Glutamine vs placebo | 2 | -2.63 (-4.99, -0.27); Moderate ^b^ | - | **-2.63 (-4.99,-0.27); Moderate** |

Note: OR, odds ratio; CoE, certainty of evidence; RD, risk difference.

a Rated down for risk of bias

b Rated down for imprecision

# Supplementary table 12. Direct, indirect and NMA estimates for duration of hospital stay (days)

| **Comparison** | **Number of trials with direct evidence, n** | **Direct estimates**  **MD (95% CI); CoE** | **Indirect estimates**  **MD (95% CI); CoE** | **NMA estimates**  **MD (95% CI); CoE** |
| --- | --- | --- | --- | --- |
|  |  |  |  |  |
| IV Glutamine vs Oral Arginine | 0 | - | 8.34 (-5.46,22.13); Moderate^a^ | 8.34 (-5.46,22.13); Low^b^ |
| IV Glutamine vs Oral Glutamine | 0 | - | 0.75 (-4.69,6.19); Moderate^a^ | 0.75 (-4.69,6.19); Low^b^ |
| IV Glutamine vs placebo | 6 | -0.46 (-3.58,2.65); High | - | -0.46 (-3.58,2.65); Moderate^b^ |
| Oral Arginine vs Oral Glutamine | 0 | - | -7.59 (-21.74,6.57); Moderate^a^ | -7.59 (-21.74,6.57); Low^b^ |
| Oral Arginine vs placebo | 1 | -8.80 (-22.23,4.63); Moderate^a^ | - | -8.80 (-22.23,4.63); Low^b^ |
| Oral Glutamine vs placebo | 4 | -1.31 (-6.30,3.40); Moderate^a^ | - | -1.21 (-5.67,3.24); Low^b^ |

Note: OR, odds ratio; CoE, certainty of evidence; RD, risk difference.

a Rated down for risk of bias

b Rated down for imprecision

# Supplementary table 13. Direct estimates for feeding intolerance

| **Comparison** | **Number of trials with direct evidence, n** | **Direct estimates**  **OR (95% CI); CoE** | **%RD (95% CI)** |
| --- | --- | --- | --- |
|  |  |  |  |
| IV Glutamine vs Placebo | 1 | 1.03 (0.83,1.30); moderate^a^ | 0.61 (-3.65, 6.08) |
| Oral Glutamine vs Placebo | 2 | **0.34 (0.19, 0.61); moderate^b^** | **-10.71 (-13.54, -6.01)** |

Note: OR, odds ratio; CoE, certainty of evidence; RD, risk difference.

a Rated down for imprecision

b Rated down for risk of bias

# Supplementary table 14. Direct estimates for weight at 37 weeks’ postnatal age or at discharge

| **Comparison** | **Trials with Direct Comparisons, n** | **Direct: MD (95% CI); CoE** |
| --- | --- | --- |
| Oral Arginine vs Placebo | 1 | 207 (-107.98, 521.98); Low^a,b^ |
| Oral Glutamine vs Placebo | 2 | 86.64 (-67.62, 240.89); Low^a,b^ |

a Rated down for risk of bias

b Rated down for imprecision

# Supplementary table 15. Incoherence analysis by the node-splitting model of all-cause mortality

| **Comparison** | **Direct** | | **Indirect** | | **Difference** | | | **τ^2^** |
| --- | --- | --- | --- | --- | --- | --- | --- | --- |
|  | **LogOR** | **SE** | **LogOR** | **SE** | **LogOR** | **SE** | **P-value** |  |
| **Placebo vs IV glutamine** | … | … | … | … | … | … | … | … |
| **Placebo vs NAC** | … | … | … | … | … | … | … | … |
| **Placebo vs oral glutamine** | -0.45 | 0.43 | 1.69 | 1.77 | -2.14 | 1.78 | 0.23 | 0.000 |
| **Placebo vs oral arginine** | -0.10 | 0.26 | 1.44 | 1.95 | -1.53 | 1.95 | 0.43 | 0.000 |
| **Oral arginine vs oral glutamine** | 0.00 | 1.04 | 0.38 | 0.55 | -0.38 | 1.18 | 0.75 | 0.000 |

# Supplementary table 16. Incoherence analysis by the node-splitting model of NEC stage II

| **Comparison** | **Direct** | | **Indirect** | | **Difference** | | | **τ^2^** |
| --- | --- | --- | --- | --- | --- | --- | --- | --- |
|  | **Coef** | **SE** | **Coef** | **SE** | **Coef** | **SE** | **P-value** |  |
| **IV glutamine vs placebo** | … | … | … | … | … | … | … | … |
| **IV NAC vs placebo** | … | … | … | … | … | … | … | … |
| **Oral arginine vs placebo** | 0.77 | 0.38 | -3.42 | 3.14 | 4.18 | 3.15 | 0.18 | 0.394 |
| **Oral arginine vs oral glutamine** | -0.86 | 1.48 | 0.25 | 0.59 | -1.11 | 1.58 | 0.48 | 0.472 |
| **Oral glutamine vs placebo** | 0.63 | 0.41 | 0.80 | 2.11 | -0.17 | 2.08 | 0.94 | 0.495 |

# Supplementary table 17. Incoherence analysis by the node-splitting model of NEC-related mortality

| **Comparison** | **Direct** | | **Indirect** | | **Difference** | | | **τ^2^** |
| --- | --- | --- | --- | --- | --- | --- | --- | --- |
|  | **Coef** | **SE** | **Coef** | **SE** | **Coef** | **SE** | **P-value** |  |
| **IV glutamine vs Placebo** | … | … | … | … | … | … | … | … |
| **Oral arginine vs Placebo** | 1.30 | 0.76 | -3.61 | 3.18 | 4.91 | 3.19 | 0.12 | 0.000 |
| **Oral arginine vs oral glutamine** | -0.60 | 1.55 | 1.96 | 1.21 | -2.56 | 2.11 | 0.23 | 0.000 |
| **Oral glutamine vs placebo** | 0.34 | 0.88 | 1.31 | 3.54 | -0.97 | 3.41 | 0.78 | 0.571 |

# Supplementary table 18. Cumulative ranking of interventions for different outcomes

| **Outcome** | **Rank** | **Intervention** | **SUCRA** | **MeanRank** |
| --- | --- | --- | --- | --- |
| **All-cause mortality** | | | | |
|  | 1 | oral arginine | 79.9 | 1.8 |
|  | 2 | oral glutamine | 56.2 | 2.8 |
|  | 3 | IV glutamine | 53.3 | 2.9 |
|  | 4 | Placebo | 44 | 3.2 |
|  | 5 | IV N-acetylcysteine | 16.6 | 4.3 |
| **NEC stage ≥II** | | | | |
|  | 1 | oral arginine | 80.6 | 1.8 |
|  | 2 | oral glutamine | 72.8 | 2.1 |
|  | 3 | IV glutamine | 47.6 | 3.1 |
|  | 4 | IV N-acetylcysteine | 26.8 | 3.9 |
|  | 5 | Placebo | 22.2 | 4.1 |
| **Culture proven late-onset sepsis** | | | | |
|  | 1 | oral glutamine | 97.1 | 1.1 |
|  | 2 | oral arginine | 41.7 | 3.3 |
|  | 3 | IV glutamine | 40 | 3.4 |
|  | 4 | Placebo | 37.4 | 3.5 |
|  | 5 | IV N-acetylcysteine | 33.9 | 3.6 |
| **NEC-related mortality** | | | | |
|  | 1 | oral arginine | 83.2 | 1.5 |
|  | 2 | oral glutamine | 45.1 | 2.6 |
|  | 3 | IV glutamine | 40.8 | 2.8 |
|  | 4 | Placebo | 30.9 | 3.1 |
| **Time to reach full enteral feed (day)** | | |  |  |
|  | 1 | oral glutamine | 91.2 | 1.2 |
|  | 2 | IV glutamine | 56.7 | 1.9 |
|  | 3 | Placebo | 2.1 | 3 |
| **Duration of hospital stay (day)** | | |  |  |
|  | 1 | oral arginine | 87.6 | 1.4 |
|  | 2 | oral glutamine | 48.5 | 2.5 |
|  | 3 | IV glutamine | 38 | 2.9 |
|  | 4 | Placebo | 26 | 3.2 |
| Note: SUCRA=surface under the cumulative ranking curve | | | |  |

# Supplementary figure 1. Plots of the SUCRAs for all-cause mortality


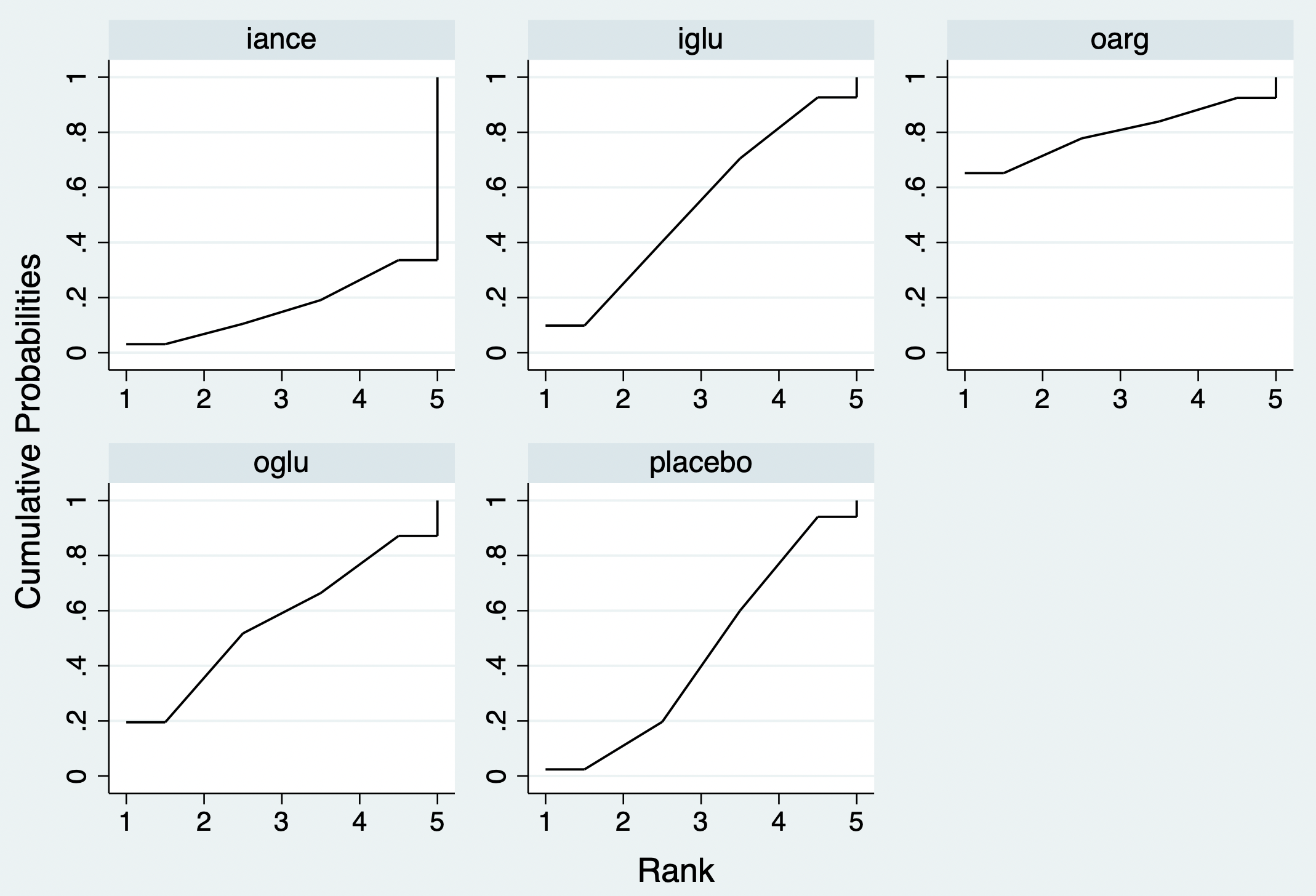


Results for the following interventions: IV N-acetylcysteine (iance), IV glutamine (iglu), Oral arginine (oarg), Oral glutamine (oglu), and placebo

# Supplementary figure 2. Plots of the SUCRAs for NEC stage II


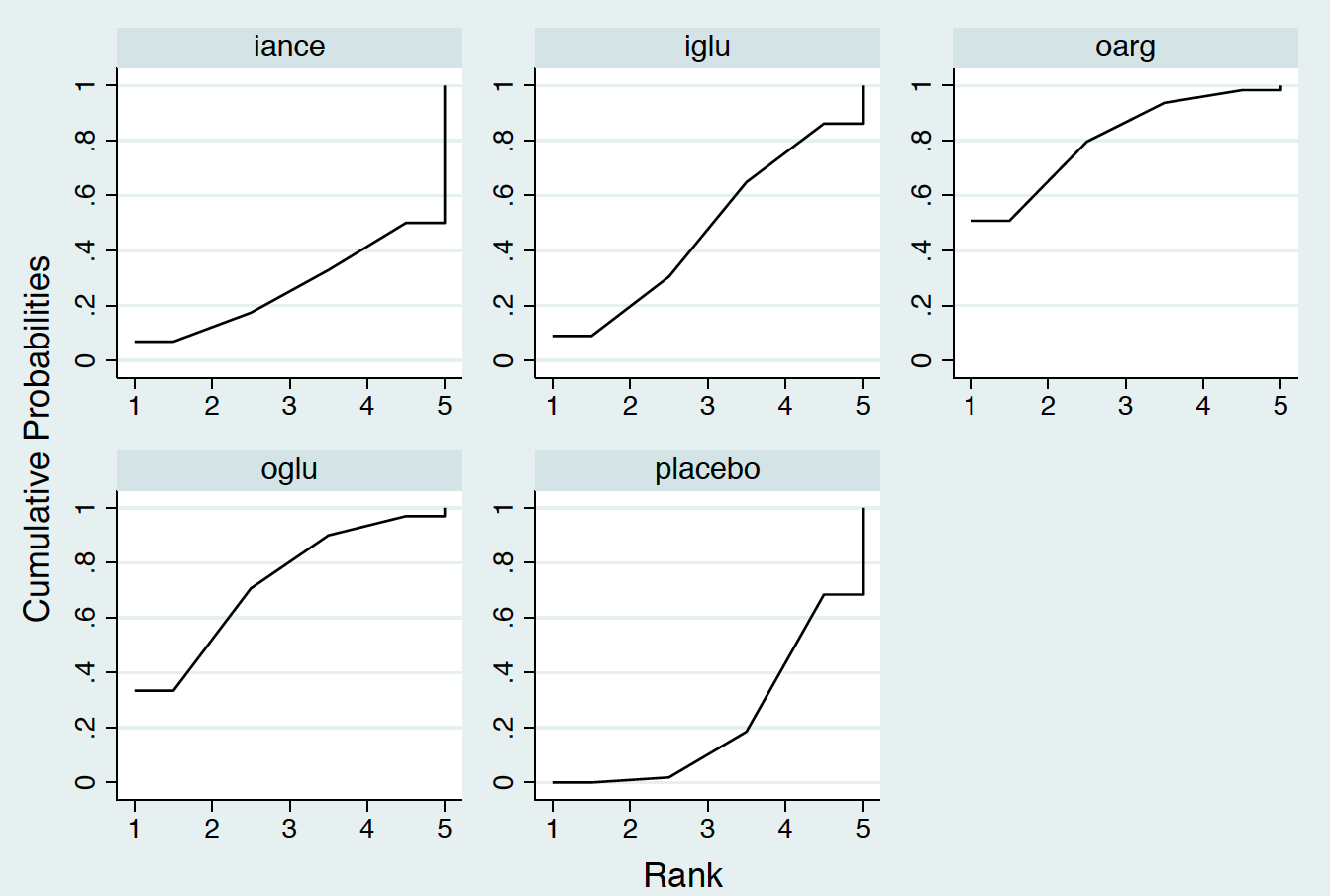


Results for the following interventions: IV N-acetylcysteine (iance), IV glutamine (iglu), Oral arginine (oarg), Oral glutamine (oglu), and placebo

# Supplementary figure 3. Plots of the SUCRAs for culture-proven Late-onset Sepsis


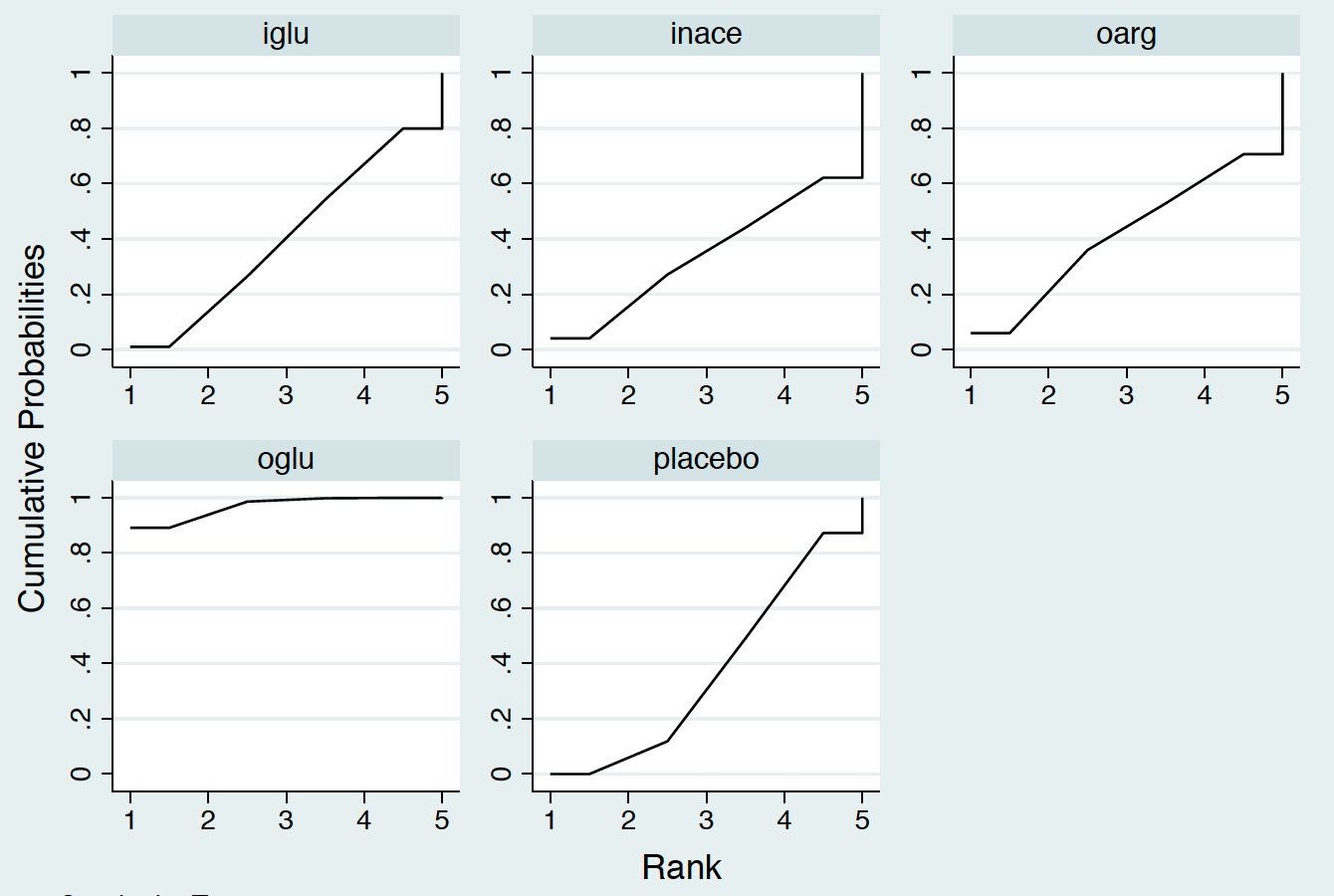


Results for the following interventions: IV N-acetylcysteine (iance), IV glutamine (iglu), Oral arginine (oarg), Oral glutamine (oglu), and placebo

# Supplementary figure 4. Plots of the SUCRAs for NEC-related mortality


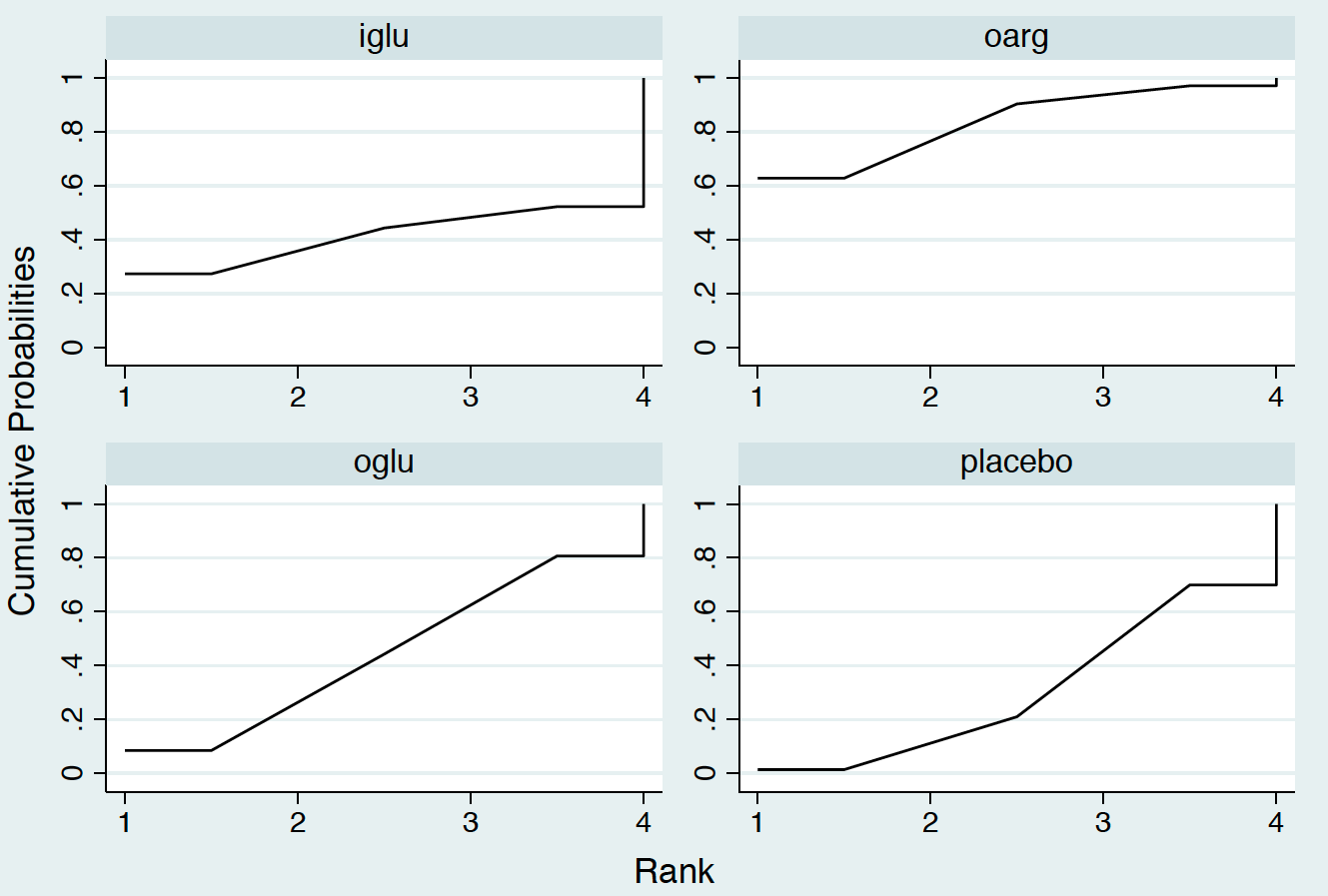


Results for the following interventions: IV glutamine (iglu), Oral arginine (oarg), Oral glutamine (oglu), and placebo

# Supplementary figure 5. Plots of the SUCRAs for time to achieve full enteral feeding (days)


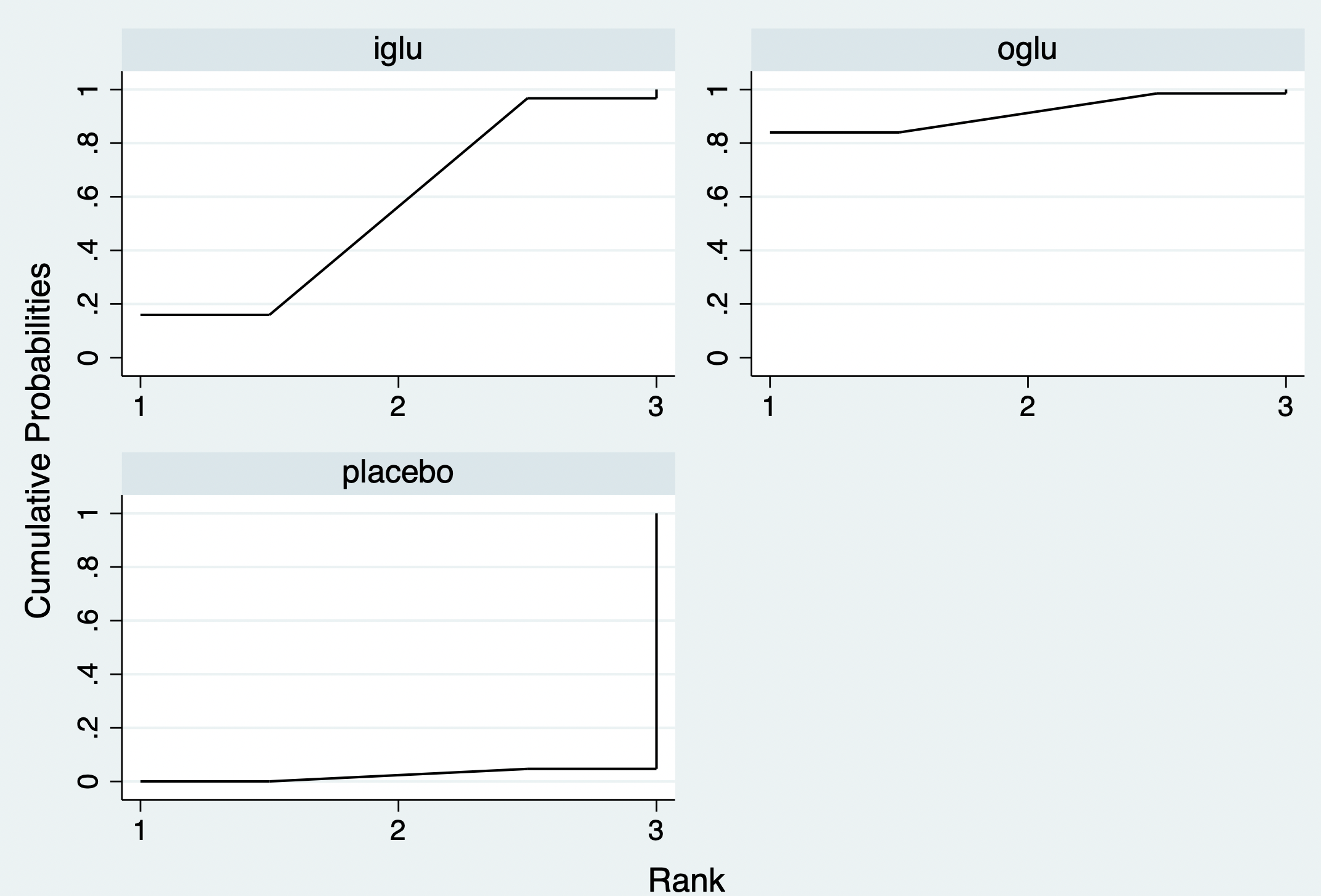


Results for the following interventions: IV glutamine (iglu), Oral glutamine (oglu), and placebo

# Supplementary figure 6. Plots of the SUCRAs for duration of hospitalization (days)


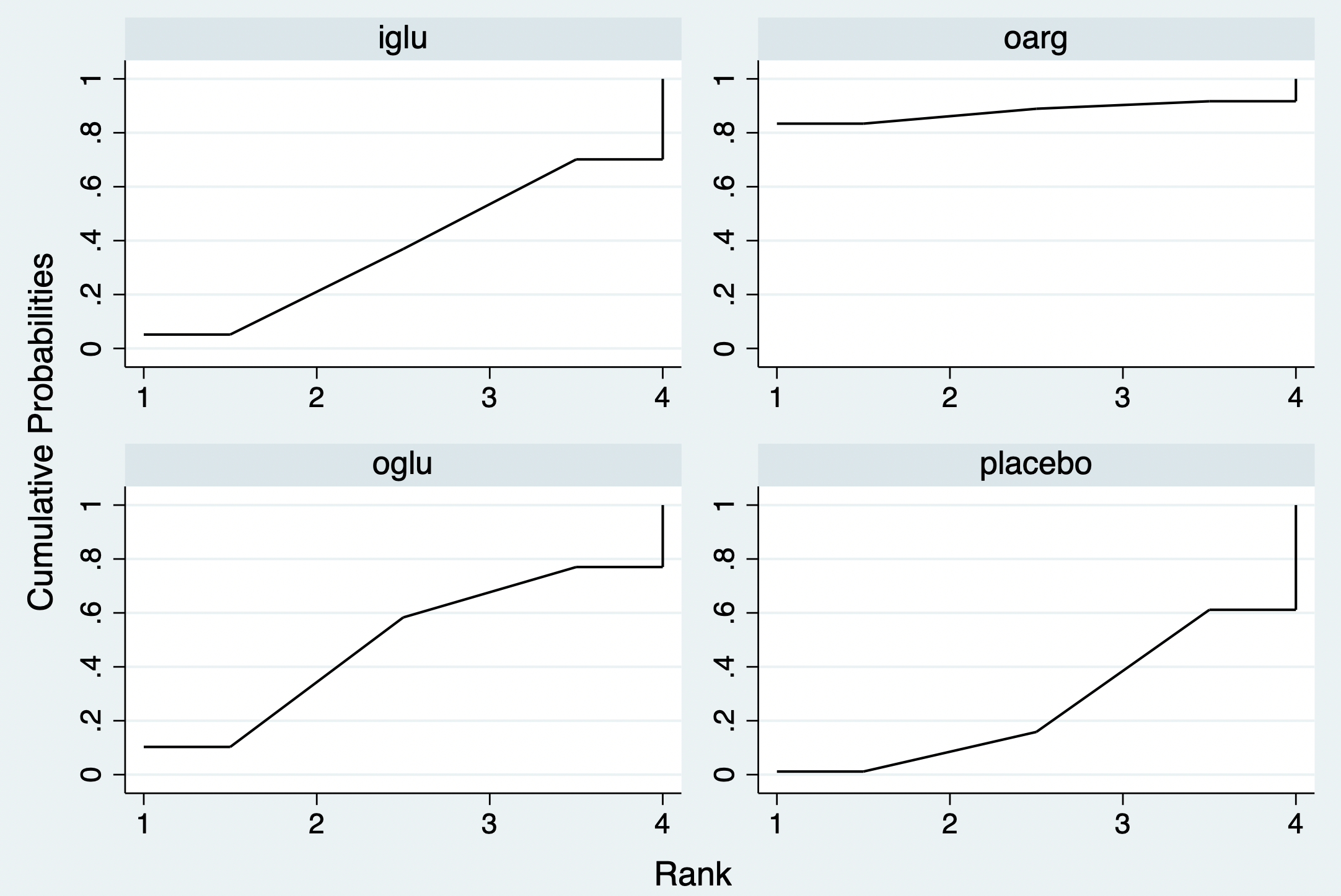


Results for the following interventions: IV glutamine (iglu), Oral arginine (oarg), Oral glutamine (oglu), and placebo
